# Mouse Retinal Organoid Growth and Maintenance in Longer-Term Culture

**DOI:** 10.3389/fcell.2021.645704

**Published:** 2021-04-27

**Authors:** Manuela Völkner, Thomas Kurth, Jana Schor, Lynn J. A. Ebner, Lara Bardtke, Cagri Kavak, Jörg Hackermüller, Mike O. Karl

**Affiliations:** ^1^German Center for Neurodegenerative Diseases (DZNE) Dresden, Dresden, Germany; ^2^Center for Molecular and Cellular Bioengineering, Technology Platform, Electron Microscopy and Histology Facility, Technische Universität Dresden, Dresden, Germany; ^3^Young Investigators Group Bioinformatics and Transcriptomics, Department Molecular Systems Biology, Helmholtz Centre for Environmental Research – UFZ, Leipzig, Germany; ^4^CRTD – Center for Regenerative Therapies Dresden, Technische Universität Dresden, Dresden, Germany

**Keywords:** organoid, retina, maturation, development, mouse, growth, sonic hedgehog, gliosis

## Abstract

Using retinal organoid systems, organ-like 3D tissues, relies implicitly on their robustness. However, essential key parameters, particularly retinal growth and longer-term culture, are still insufficiently defined. Here, we hypothesize that a previously optimized protocol for high yield of evenly-sized mouse retinal organoids with low variability facilitates assessment of such parameters. We demonstrate that these organoids reliably complete retinogenesis, and can be maintained at least up to 60 days in culture. During this time, the organoids continue to mature on a molecular and (ultra)structural level: They develop photoreceptor outer segments and synapses, transiently maintain its cell composition for about 5–10 days after completing retinogenesis, and subsequently develop pathologic changes – mainly of the inner but also outer retina and reactive gliosis. To test whether this organoid system provides experimental access to the retina during and upon completion of development, we defined and stimulated organoid growth by activating sonic hedgehog signaling, which in patients and mice *in vivo* with a congenital defect leads to enlarged eyes. Here, a sonic hedgehog signaling activator increased retinal epithelia length in the organoid system when applied during but not after completion of development. This experimentally supports organoid maturation, stability, and experimental reproducibility in this organoid system, and provides a potential enlarged retina pathology model, as well as a protocol for producing larger organoids. Together, our study advances the understanding of retinal growth, maturation, and maintenance, and further optimizes the organoid system for future utilization.

## Introduction

Retinal degenerative diseases affect millions of patients worldwide, and for the majority there are no effective therapies (Scholl et al., 2016; Flaxman et al., 2017). Animal disease model research continuously increases our understanding of pathomechanisms, reveals potential therapeutic targets, and is successfully used to develop gene, drug, and cell therapies (Veleri et al., 2015). However, many drug-discovery and testing studies are still limited, since these require very large numbers of animals or retinas for cell culture. Very few human donor tissues are available, and these are of variable quality and also usually only available at disease endstage. Larger-scale animal studies, particularly for large animal models, have ethical and practical limitations. Further, the translation from animals to human patients faces various issues which might be overcome with access to an unlimited number of mouse and human retinal organoids (Kruczek and Swaroop, 2020). Organoid systems might reduce the number of animals required, for example using mouse retinal organoids (MROs) in combination with matching mouse *in vivo* models, as well as with related preclinical human retinal organoid models.

Mouse and human pluripotent stem cell derived retinal organoid systems make the self-organized development of 3D, stratified retinal tissues possible, theoretically in unlimited numbers (Bell et al., 2020; Kruczek and Swaroop, 2020; O’Hara-Wright and Gonzalez-Cordero, 2020). Retinal organoid research has been shown to facilitate basic and translational research, like studies of retinogenesis, physiology, disease mechanisms, and preclinical studies of cell- and gene-based therapies. However, the requirements for reliably and stably reproducing all structural and functional properties of the physiological retina in the organoid system, as well as those facilitating its long-term maintenance in culture in the mouse and human system, have not yet been fully defined: No organoids have been systematically assessed beyond neonatal stages yet. It is particularly important to stably emulate mature retinas for studies of visual function, and for modeling pathologies with progression or onset after the neonatal stage: this includes most inherited retinal dystrophies and age-related macular degeneration. The first models reproducing genetic defects, like deficits in photoreceptor-specific proteins, cilia, and photoreceptor dystrophy, have been reported in human organoids (Parfitt et al., 2016; Deng et al., 2018; Gantner et al., 2019; Huang et al., 2019; Lane et al., 2020), and in MRO (Mookherjee et al., 2018). Generally, it has not yet been possible to reproduce complex pathologies with several distinct processes, like neurodegeneration, glial pathologies, and neural remodeling. So far, one study has experimentally modeled induced photoreceptor degeneration in MROs, but analysis of longer-term pathogenesis and therapy was limited by spontaneous degeneration in the controls (Ito et al., 2017). In general, the organoid properties required for effective modeling of retinal pathologies, and those currently provided by organoid systems, are still incompletely understood and defined.

Several studies so far could show that retinogenesis, which describes the process of multipotent stem cells dividing and differentiating into all major types of retinal neurons, as well as Müller glia, is reproduced in mouse retina organoids (Eiraku et al., 2011; Gonzalez-Cordero et al., 2013; Decembrini et al., 2014, 2020; Hiler et al., 2015; Chen et al., 2016; Völkner et al., 2016; Ito et al., 2017; DiStefano et al., 2018; Ueda et al., 2018; Brooks et al., 2019). The depletion of stem cells, and thus the completed differentiation (cell birth), of retinal cells marks the timepoint in retinogenesis when all retinal cells are postmitotic, and the retina is therefore also described as postmitotic. Retinal cell birth is complete at about postnatal day (P) P5–P6 in the central region, and at P10 in the periphery of the mouse retina *in vivo* (Young, 1985; Ogawa et al., 2017). Young mice open their eyes, and the retina becomes functional, at about P12, although it fully matures until about P30. Various MRO protocols and modifications reproduce 3D-like retinas in culture, and offer advantages for specific requirements and applications, and for more efficient organoid production. Retinal organoids undergo the same major developmental changes as in the mouse *in vivo* (Eiraku and Sasai, 2011; Eiraku et al., 2011; Gonzalez-Cordero et al., 2013; Decembrini et al., 2014; Hiler et al., 2015; Chen et al., 2016; Völkner et al., 2016; Ito et al., 2017; DiStefano et al., 2018; Ueda et al., 2018; Brooks et al., 2019; Decembrini et al., 2020). However, it has not yet been possible to mature and maintain MROs to the adult state. There may be differences in MRO size, timing of retinogenesis, cell composition and organoid viability and stability might vary, depending on the protocol. This may limit retinal phenotype stability, longer-term culture, and application potentials. Notably, MROs have been studied up to the end of retinogenesis, or slightly thereafter (Eiraku and Sasai, 2011; Eiraku et al., 2011; Decembrini et al., 2014; Hiler et al., 2015; Chen et al., 2016; Völkner et al., 2016; Ito et al., 2017; DiStefano et al., 2018; Ueda et al., 2018; Brooks et al., 2019; Decembrini et al., 2020), which ranges between D21 and D35: most studies report that organoids did not survive long after that. Thus, longer-term culture of MROs has not yet been systematically assessed within or between protocols. For example, some protocols may provide high MRO yields (Gonzalez-Cordero et al., 2013; Decembrini et al., 2014), but develop with intertwined retinal and non-retinal structures. MROs may develop with deficits of the inner retina, although bioreactor culture and supplements may improve this (Hiler et al., 2015; Chen et al., 2016; DiStefano et al., 2018; Brooks et al., 2019). Further, organoid neuroepithelial developed into eyefields in the pioneering protocol, but infrequently form optic vesicles with most mESC lines (Hiler et al., 2015; Völkner et al., 2016). To achieve efficient generation of complete, 3D, stratified retinas, we devised the trisection protocol. This yields three evenly-sized, smaller neuroepithelial parts developing into twice as many MROs per starting aggregate. These show very low variances in several aspects at cellular and molecular levels, which means they are easier to use (Völkner et al., 2016; Santos-Ferreira et al., 2019; Völkner et al., 2019). Further, O_2_ and nutrient supply in larger MROs might limit proper development and homeostasis (DiStefano et al., 2018), and thus smaller MROs might be advantageous. This prompted us to hypothesize that MROs developed using the trisection method might also provide reproducible and stable properties beyond the completion of retinogenesis, e.g., facilitating further maturation and survival in longer-term culture.

In this study, we assessed retinal maturation and stability in longer-term culture up to D60 in the trisection-based MRO system. We previously demonstrated that MROs reproducibly develop within 20 days. Here, we show that MROs reproducibly complete retinal cell generation and continue to mature while maintaining comparable cell numbers over a transient timeframe, and subsequently develop some pathologic changes, particularly, in the inner but also outer retina. To experimentally validate and apply this MRO system, we tested stimulation of retinal growth and found that growth becomes limited with increasing organoid age. Thus, we provide a modified protocol for the efficient and reproducible production of larger MROs, which increases the cell yield and possibly provides an organoid pathology model for congenital retinal enlargement. In summary, our data confirm that this MRO system provides experimental access to embryonic, neonatal, and postmitotic retinas, and opens up various application potentials.

## Materials and Methods

### mESC Maintenance

E14TG2a (MMRRC, UC Davis) mouse embryonic stem cells (mESC) were cultured in mESC medium (DMEM, 15% FBS, 1% pyruvate, 1% NEAA, 1% GlutaMAX, 1% penicillin/streptomycin, 1 mM 2-mercaptoethanol) supplemented with 10^3^ U/ml LIF and 1 μM PD0325901 on 10 cm tissue-culture plates (BD Falcon). Cells were passaged every 2 days using TrypLE Express (Invitrogen) and reseeded at a density of 1 × 10^6^ cells/plate.

### Mouse Retinal Organoid Culture

Mouse retinal organoids (MROs) were differentiated using the trisection protocol (TRIP) as previously described (Völkner et al., 2016, 2019). Briefly, mESCs were dissociated to single cells using TrypLE Express (Invitrogen), and plated into 96-well low-adhesion plates (U-bottom, Lipidure Coat, NOF) at 3000 cells per well in retinal differentiation medium (GMEM, 1% penicillin/streptomycin, 1% NEAA, 1% pyruvate, 1.5% KnockOut serum replacement, 1 mM 2-mercaptoethanol). On day (D) 1, 2% Matrigel (growth-factor reduced, BD Biosciences) was added to the culture media. On D7, MROs were transferred to bacterial-grade petri dishes (Greiner Bio-One) and further cultured (40% O_2_) in DMEM/F12 with GlutaMAX, supplemented with 1% N-2 and 1% penicillin/streptomycin. On D10, MROs were manually trisected using surgical tweezers (Fine Science Tools, Dumont No. 5) and further cultured in DMEM/F12 with GlutaMAX, 1% N-2 supplement, 1% penicillin/streptomycin, 10% FBS in bacterial-grade petri dishes. Synthetic retinoid analog EC23 (0.3 μM) was added from D10 to D14. Half of the media was exchanged every other day thereafter.

In some sets of experiments, smoothened agonist (SAG, Enzo Life Sciences) was added to the culture from D14–D21, D20–D25, or D25–D30. SAG was added at a final concentration of 0.25 μM and replaced at each media change (every second day). SAG was dissolved in sterile DMSO and an equal amount of DMSO (0.25 μl/ml) was applied to control MROs. To assess if SAG treated MRO develop all retinal cell types, MRO were treated with SAG from D14-21, then SAG was removed by a full media change, and MRO were kept in culture for 5 more days and analyzed at D26.

To assess MRO stability in different protocols, MROs were differentiated from the same batch of mESC cells in parallel using the trisection (see above) and mother-organoid protocols (MOP), respectively. MRO generation by the mother-organoid approach was performed as previously described (Gonzalez-Cordero et al., 2013; Santos-Ferreira et al., 2016). Briefly, mESCs were treated in the same way as for the trisection protocol (see above) until D7. Then, developing MROs were kept in the 96-well plate for an additional 2 days and transferred to bacterial-grade petri dishes (Greiner Bio-One) on D9. In contrast to the trisection protocol, MROs were not cut, but were further cultured under the same conditions (37°C, 20% O_2_) in DMEM/F12 with GlutaMAX, supplemented with 1% N-2 and 1% penicillin/streptomycin. EC23 (0.3 μM) was added to the culture medium from D14 onward, and half of the media was exchanged every other day thereafter.

### Mouse Retinal Explant Culture

Mouse retinal explant culture was performed as previously described (Löffler et al., 2015). Briefly, at postnatal day (P) 10, retinas were dissected in HBSS and placed with the outer nuclear layer (ONL) facing down on Millicell cell culture inserts (Millipore). Cell culture inserts were placed in 6-well plates with 1ml of culture medium [DMEM F12, (US Biologicals, D9807-05), 1% N-2, 5 mM Hepes, 1% penicillin/streptomycin, 1% FBS, 1 mM L-glutamine, 0.6% D + glucose, 0.2% NaHCO_3_]. Recombinant human EGF (R&D) was added to the culture medium daily at 50 ng/ml and half of the medium was replaced daily. Retinas were cultured at 5% CO_2_ and 37°C. Mice used in this study were C57BL/6JRj^1^. Animals were maintained under a 12 h light/dark cycle with access to food and water *ad libitum* under specified pathogen-free conditions. All necessary licenses were obtained according to the TU Dresden and German Federal regulations (approved by Landesdirektion Dresden, Germany). Research followed the guidelines of the ARVO Statement for the Use of Animals in Ophthalmic and Visual Research.

### Immunohistochemistry

For immunohistochemistry, tissue was fixed in 4% PFA in PBS, cryoprotected in a graded series of sucrose solutions and embedded in OCT compound (Sakura Finetek). Tissue was cut into 12 μm sections, mounted on Superfrost Ultra Plus slides (Thermo Scientific) and stored at –80°C. Sections were washed in PBS for 15 min then, if necessary, antigens were retrieved via citrate (10 mM sodium citrate, pH 6.0, 30 min at 70°C). The tissue was blocked for 30 min at RT in blocking solution (0.5% BSA, 0.3% Triton-X-100 in PBS), followed by primary-antibody incubation (48 h, 4°C). Tissue was washed in PBS (3×, 10 min) and species-specific secondary antibodies conjugated to fluorophores (488, Cy3, 649; Dianova, 1:1000) were applied for 1 h at RT. Nuclei were stained by DAPI (AppliChem). Tissue was washed again in PBS and coverslipped using Fluoromount-G (Southern Biotechnology). Filamentous actin was visualized using Phalloidin488 staining (PD, Invitrogen; 1:500, 15 min at RT) after secondary-antibody incubation. TUNEL assay for cell death analysis was performed before primary antibody incubation using *In Situ* Cell Death Detection Kit TMR red (Sigma-Aldrich, Roche products) according to the manufacturer’s instructions. Primary antibodies used in this study are listed in Supplementary Table 1.

### Qualitative and Quantitative Imaging-Based Analysis

Samples were imaged on a Zeiss ApoTome2 or Zeiss Spinning Disk confocal microscope. For cell counts, 100 μm-wide regions of interest (ROI) were used. The *x*-axis of each ROI was positioned radially to the organoid center, and the *y*-axis aligned perpendicular to the organoid surface. The height of the ROI was set to include the entire epithelial width. ROI images are *z*-axis projections of 5 × 1 μm, i.e., five planes, 1 μm apart acquired in Apotome mode using a 20 × Plan-Apochromate objective. For cell counts, images were 3D reconstructed (maximum intensity projection) and counted using Fiji (Schindelin et al., 2012). For quantitative analysis of RHO, PDE6B, GNAT1, BSN, SYP, and GFAP, images were thresholded in Fiji (mean threshold mode) and the pixel area above the threshold was measured and normalized to ROI or DAPI area. Phospho-histone-3 (PHH3)-labeled cells, as well as rosettes, were counted per entire organoid section, and normalized to each organoid circumference. Organoid circumference and epithelial thickness were measured on microscopic images of entire central organoid sections using Fiji. For cell death analysis of developing MRO, ROI were automatically thresholded in Fiji (mean threshold mode, separately for each channel), and pixel area above threshold was measured and normalized to DAPI pixel area. To discriminate between cell death in ONL-like and inner retinal layers, ROI were sub-divided in an outer (ONL) and inner (INL/GCL) layer based on ELAVL3/4 staining from D15 onward. For qualitative analysis of marker expression, at least 10 MROs from *N* ≥ 1 independent experiments were assessed.

### Histology of Methacrylate Resin Sections

Histology of MRO was performed as previously described (Völkner et al., 2019). Briefly, MRO were fixed in modified Karnovsky’s fixative (2% glutaraldehyde, 2% paraformaldehyde in 50 mM HEPES) overnight at 4°C (Kurth et al., 2010). Samples were washed, postfixed in 1% OsO_4_/PBS, washed again and dehydrated in a graded series of ethanol. Samples were infiltrated in Technovit 7100 and embedded. Sections were cut at 2 μm using a rotary microtome. Sections were stained with 1% toluidine blue/0.5% borax and imaged using the Keyence Biozero 8000 fluorescence microscope.

### Transmission Electron Microscopy (TEM)

Transmission electron microscopy of MROs was performed as previously described (Völkner et al., 2019). Briefly, MROs were fixed in 4% formaldehyde (prepared from paraformaldehyde prills) in 100 mM phosphate buffer and dissected for different applications. Samples selected for resin embedding and TEM were postfixed in modified Karnovsky’s fixative (2% glutaraldehyde, 2% paraformaldehyde in 50 mM HEPES) overnight at 4°C (Kurth et al., 2010). Samples were washed and further postfixed in 2% aqueous OsO_4_ solution containing 1.5% potassium ferrocyanide and 2 mM CaCl_2_. After washing, samples were incubated in 1% thiocarbohydrazide, washed again, and contrasted in 2% aqueous OsO_4_ for a second time. After washing, samples were *en-bloc* contrasted with 1% uranyl acetate/water, washed again in water, dehydrated in a graded ethanol series and infiltrated in the epon substitute EMBed 812. After embedding, samples were cured at 65°C overnight. Ultrathin sections were cut with a Leica UC6 ultramicrotome and collected on formvar-coated slot grids. Sections were stained with lead citrate (Venable and Coggeshall, 1965) and uranyl acetate, and imaged on a FEI Morgagni D268 (camera: MegaView III, Olympus) or a Jeol JEM1400 Plus (camera: Ruby, JEOL) both running at 80 kV acceleration voltage.

### Correlative Light Electron Microscopy (CLEM) of Ultrathin Cryosections

To perform CLEM, small pieces of dissected MROs were fixed in 4% paraformaldehyde (PFA) in 0.1M phosphate buffer (PB, pH 7.4) and processed for Tokuyasu cryo-sectioning (Tokuyasu, 1980; Slot and Geuze, 2007). Samples were washed in PB, infiltrated stepwise into 10% gelatin at 37°C, cooled down on ice, incubated in 2.3M sucrose/water at 4°C, mounted on pins (Leica # 16701950), and plunge-frozen in liquid nitrogen. 70–100 nm sections were cut on a Leica UC6 + FC6 cryo-ultramicrotome and picked up in methyl cellulose/sucrose [1:1 2% methyl cellulose (MC, Sigma M-6385, 25 centipoises) and 2.3M sucrose].

To facilitate the identification of photoreceptors by CLEM, sections were stained with antibodies for RHO or RCVRN (Fabig et al., 2012; Santos-Ferreira et al., 2016). In brief, grids were incubated in PBS at 37°C for 20 min, washed with 0.1% glycin/PBS, blocked with 1% BSA/PBS and incubated with the primary antibodies for 1 h. For primary antibodies raised in rabbit, grids were washed in PBS and incubated directly with protein A conjugated to 10 nm gold particles for 1 h, washed again in PBS and post-fixed in 1% glutaraldehyde (5 min). For mouse primary antibodies, sections were incubated with rabbit-anti-mouse bridging antibodies (Slot and Geuze, 2007), followed by protein A gold and postfixation. Subsequently, sections were incubated with fluorescently-labeled secondary antibodies (goat-anti-rabbit or goat-anti-mouse Alexa488), washed with PBS, stained with DAPI and washed in water. Grids were mounted in 50% glycerol/water between two coverslips, and imaged with the Keyence Biozero 8000 fluorescence microscope. Sections were demounted, washed with distilled water, stained with neutral uranyl oxalate [2% uranylacetate (UA) in 0.15 M oxalic acid, pH 7.0], washed in water, and incubated in MC containing 0.4% UA for 5 min. Grids were looped out, air dried and sections were analyzed on a JEM 1400Plus transmission electron microscope at 80 kV. Images from the exact same sections and regions as for fluorescence microscopy were taken with a Ruby digital camera (JEOL). Overlays of fluorescent and TEM images for CLEM were prepared using Fiji and Adobe Photoshop by overlaying structures clearly distinguishable both in fluorescence and transmission electron microscopic images, i.e., cell nuclei and junctions.

### Scanning Electron Microscopy (SEM)

Mouse retinal organoids were fixed in modified Karnovsky’s fixative (2% glutaraldehyde, 2% paraformaldehyde in 50 mM HEPES). After washing in HEPES and PBS, samples were postfixed in 1% osmium tetroxide/PBS, washed in PBS and water, and dehydrated in a graded ethanol series. Samples were critical-point dried using the Leica CPD 300 (Leica Microsystems, Vienna, Austria). Dried whole MROs were mounted on 12 mm aluminum stubs; some MROs were manually dissected using a scalpel. This way, the samples break apart, preferentially between cell borders. Finally, samples were sputter-coated with gold using the Baltec SCD 050 (Leica) and analyzed with a JSM 7500F cold field emission SEM (JEOL) at 8 mm working distance and 5–10 kV acceleration voltage using the lower secondary electron detector.

### Statistical Analysis

Statistical analysis was performed with GraphPad Prism 8 software using one-way ANOVA (Tukey’s *post hoc* test) or Student’s unpaired *t*-test. Results were considered significant for *p* < 0.05, and data were plotted as mean ± standard deviation (SD). Standard deviations were computed for total organoid numbers (n) from N experiments. *N* ≥ 3 independent experiments with *n* ≥ 5 MROs per experiment were analyzed for each dataset, unless differently indicated in the figure legend.

### Transcriptome Analysis

#### Library Preparation and Transcriptome-Sequencing

For transcriptome analysis, MROs were sampled at D20, 22, 25, and 30 (*n* = 6 MRO each, *N* = 1 independent experiment). Individual organoids were lysed in 100 μl TRIzol by mortar-pestle homogenization, RNA was isolated using miRNeasy Kit (Qiagen) and Maxtract High Density tubes (Qiagen), residual DNA was removed using Ambion TURBO DNA-free Kit (Thermo Fisher) and RNA cleaned up by ethanol-precipitation. RNA concentration was determined by Qubit 2.0 instrument using the Quant-iT RNA kit (Thermo Fisher Scientific). The RNA integrity for each sample was controlled with the RNA 6000 Nano Assay and the Agilent 2100 Bioanalyzer (Agilent Technologies). All samples included in the experiment had RIN > 8, and 100 ng total RNA was used for rRNA depletion. Ribosomal RNAs were removed from total RNA using the Ribo-Zero Gold H/M/R Magnetic Kit (Illumina). A strand-specific library for transcriptome sequencing was prepared using the ScripSeqv2 Kit (Illumina), which was checked by Agilent 2100 Bioanalyzer system with a High Sensitivity DNA Kit (Agilent). Library concentration was determined by Qubit 2.0 instrument using the Quant-iT dsDNA High Sensitivity kit (Thermo Fisher Scientific). Forty nanogram from each library was pooled. Library pool was size selected in a range of 200–600 bp using preparative agarose gel in combination with MinElute Gel Extraction Kit (Qiagen). Single-end sequencing with 30 million reads per sample was performed at a length of 75 bases on HiSeq2500 (Illumina).

### Analysis of RNA-Seq Data

The data processing prior to its analysis was performed in uap (Kampf et al., 2019). Sequencing reads were demultiplexed using bcl2fastq (Illumina, release 2.20) and sequencing adapters trimmed using cutadapt (release 1.5) (Martin, 2011). Quality control was performed using the fastqc program (release 0.11.2) and fastx-toolkit (release 0.013). Reads were aligned to the mouse genome (UCSC Mouse Dec. 2011 GRCm38/mm10 Assembly) using hisat2 (release 2.1.0) (Kim et al., 2015) and subsequently sorted by name and genomic location using samtools (release 1.1) (Li et al., 2009). New transcripts were generated using stringtie (release 1.3.3, reference annotation: Gencode v26) (Pertea et al., 2015), and sample-wise assemblies were merged applying stringtie-merge. Subsequently, new transcripts were annotated with respect to their relative genomic position to known genes using cuffcompare (2.2.1) (Trapnell et al., 2010). Transcripts entirely located within a reference intron, were considered intergenic and those overlapping an exon of a known gene on the opposite strand were kept for further analysis. The number of reads overlapping a known gene from the reference annotation or a new transcript was counted using htseq-count (0.6.1) (Anders et al., 2015). The subsequent data analysis was mainly performed using the R language for statistical computing (version 3.5.1). Differential gene expression analysis was performed using Bioconductors DESeq2 R package (version 1.22.1, Love et al., 2014). The Variance-mean dependence was estimated in our count data, and the samples from each timepoint were tested against each other for differential expression using the negative binomial distribution. Genes with a base mean below 10 were discarded, and for all remaining genes the *p*-value was adjusted using Benjamini and Hochberg adjustment method (Haynes, 2013). All genes with an adjusted *p*-value below 0.01 (=FDR) were regarded as differentially expressed. The *z*-score is calculated by subtracting the population mean from a given data point and divided by the standard deviation. Gene set enrichment analysis was performed on the DE genes using Bioconductors Ensemble of Gene Set Enrichment Analysis (EGSEA) R package (Martin, 2011). We included all available gene sets (h, c1, c2, c3, c4, c5, c6, c7) from MSigDB (Haynes, 2013), as well as gene sets from GeneSetDB (Araki et al., 2012). The top 20 gene sets were kept and clustered according to the expression profile of the underlying genes. For further analysis, we used custom-made Genes of interest (GOI) lists based on data in the literature and integrated with the results of the differential expression analysis by mapping the gene identifiers (ensemble gene ID).

### Image Processing and Figure Preparation

Graphs and schematic illustrations were prepared using GraphPad Prism 8 and Adobe CS Illustrator 2020 software, respectively. Images were optimized, making minor changes to contrast, and cropped in Adobe CS Photoshop 2020 and arranged using Adobe CS Illustrator 2020.

## Results

### Retinal Organoid Retinogenesis and Maintenance in Longer-Term Culture

**FIGURE 1:**
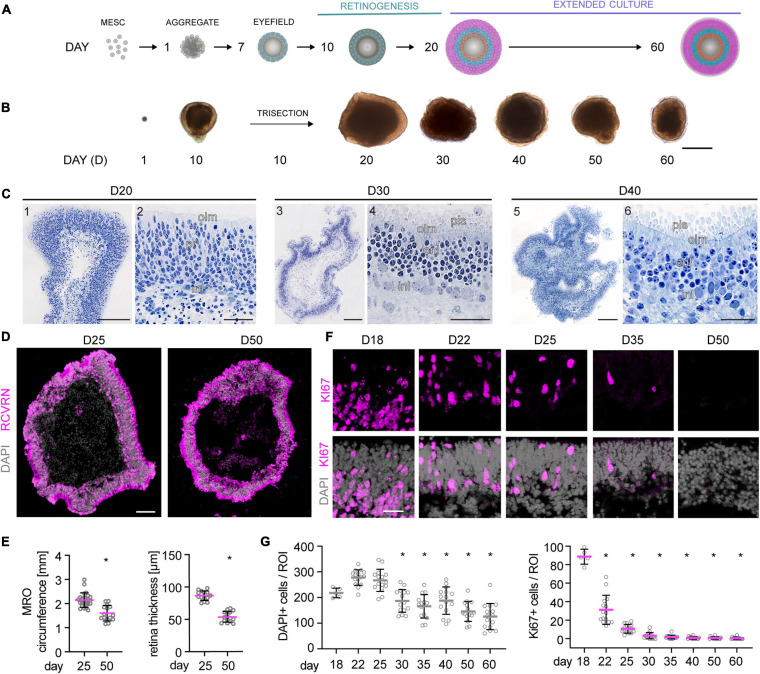
Morpho-histological characterization of MROs within longer-term culture. **(A)** Schematic of the experimental approach. Mouse retina organoids (MROs) were differentiated as previously described (Völkner et al., 2016, 2019) and analyzed at different timepoints in extended culture after the end of retinogenesis (postmitotic). **(B)** Representative phase contrast images of MROs at different stages of differentiation, showing organoid growth during the first 20 days of culture. Upon extended culture, organoid size decreases and epithelia become less optically translucent. **(C)** Histology of methacrylate resin embedded MROs. **(C1,2)** D20, overview **(1)** and detail **(2)** showing a regular neuroepithelial layer with photoreceptors (pr) and interneurons (in). **(C3,4)** D30, overview **(3)** and detail **(4)** showing MRO with well-developed inner and outer nuclear layers (inl, onl), outer limiting membrane (olm) and photoreceptor inner segment (pis). **(C5,6)** D40, overview **(5)** and detail **(6)** showing photoreceptors with well-defined inner segments, a clear olm, but less clear border between outer and inner nuclear layers. **(D)** Overview image of organoid cryosections at D25 and D50 stained for RCVRN (photoreceptors) and DAPI. MROs decrease in size and the epithelia appear thinner at D50. **(E)** Quantification of MRO circumference and epithelial thickness on retinal cryosections. *p < 0.0001 (Student’s *t*-test). **(F–G)** Representative images and quantification of cell cycle marker KI67 and DAPI in MRO in extended culture. At D18, KI67 is still detected in many cells, but it becomes rapidly reduced thereafter, suggesting MRO become and remain postmitotic. The total cell number (DAPI) per region of interest (ROI) decreases from D30, suggesting cell loss. MRO were derived from (N) independent differentiations: D18 *N* = 1, all others *N* = 3. *p < 0.0001 (ANOVA). D, day. Scale bars: **(B)** 500 μm; **(C)** 1, 3, 5: 200 μm, and 2, 4, 6: 50 μm; **(D)** 100 μm; **(F)** 25 μm.

**FIGURE 2:**
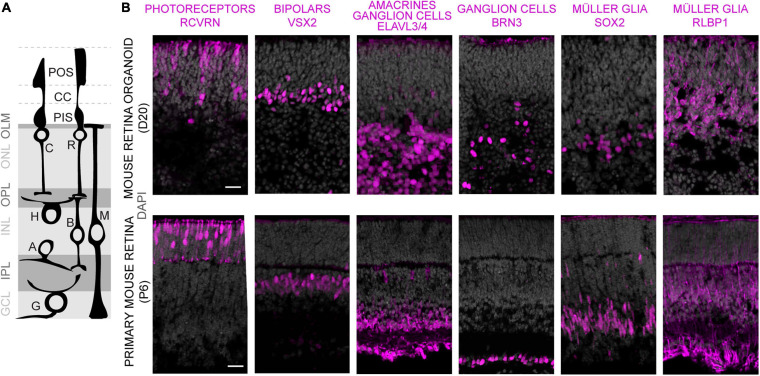
Characterization of retinal cell composition in MROs. **(A)** Schematics showing major structure and cell types of the vertebrate retina. Photoreceptors: C, cone; R, rod. Interneurons: B, bipolars; A, amacrines; H, horizontals; G, ganglion cells. MG, Müller glia. Photoreceptor cell structures: CC, connecting cilium; PIS, photoreceptor inner segment; POS, photoreceptor outer segment; OLM, outer limiting membrane. Retinal layers: ONL/INL, outer/inner nuclear layers; GCL, ganglion cell layer; OPL/IPL, outer/inner plexiform layers. **(B)** Comparison of MRO at day (D) 20 with primary mouse retina from postnatal day (P) 6 by immunostaining analysis of cryosections using established markers. Scale bars: 25 μm.

To evaluate whether MROs can be maintained beyond the end of retinogenesis, we utilized our previously-developed trisection protocol optimized for MRO generation (Völkner et al., 2016, 2019). Here, we hypothesized that the trisection system might facilitate further MRO maturation and maintenance in longer-term culture due to its low variance in development. To assess this, MROs were generated and sampled at increasing ages (Figure 1A), days (D) 18, 22, 25, 30, 35, 40, 50, and 60 (unless noted otherwise: *N* ≥ 3 independent experiments with *n* ≥ 5 MROs per experiment and timepoint). Phase contrast live imaging of developing whole MROs reproducibly showed growing and bright retinal epithelia at least up to D25 (Figure 1B). Thereafter, epithelial brightness and MRO size slowly decreased with increasing age, although we did not observe catastrophic tissue deterioration. Thus, we reproducibly maintained MROs longer than previously reported by any other MRO system. Qualitative analysis by histology (Figure 1C) and immunostaining of MRO sections (Figure 1D) showed that a stratified retinal epithelium and a large outer retinal layer with RCVRN+ photoreceptors were maintained up to D50. Quantitative analyses confirmed that MROs showed a low variance in size and epithelial thickness (Figure 1E), and an age-dependent reduction in size and epithelial thickness to 74% and 62% of the maximum, respectively. Total cell counts based on the nuclei stain DAPI on MRO sections (Figures 1F,G) showed that the cell number remained stable until D25 (267 ± 43 DAPI cells/ROI), started to decline at D30 (187 ± 45 DAPI cells/ROI), and further decreased slowly until D60 (126 ± 51 DAPI cells/ROI). Next, we determined whether MROs in our system not only complete retinal cell generation (i.e., stem cells differentiate into retinal neurons and glia, Völkner et al., 2016), but also whether MROs maintain a postmitotic state. For example, degenerating neurons may reactivate cell-cycle machinery *in vivo* (Zencak et al., 2013), and glia may undergo pathologic proliferative gliosis in culture and *in vivo* (Karl et al., 2008; Löffler et al., 2015; Sardar Pasha et al., 2017). KI67 is expressed in all phases of the cell cycle in stem cells of the developing retina, and transiently maintained in early postmitotic retinal cells (Pacal and Bremner, 2012) like other stem-cell and cell-cycle related genes (Blackshaw et al., 2004). Quantification of immunolabeled KI67+ cells on MRO sections at all sampled timepoints (Figures 1F,G) showed an age-dependent and rapid decline between D18 and D25, and KI67 was rarely detectable thereafter. These data support and extend our previous findings based on mitosis markers, birthdating, and gene expression changes of retinal stem cells, neurons, and glia (Völkner et al., 2016). To further characterize retinal cell types in MROs, we performed side-by-side immunostainings with primary mouse retinas (Figures 2A,B and Supplementary Figure 1), including markers for photoreceptors (RCVRN), bipolars (VSX2, PROX1), amacrines, horizontals, and ganglion cells (ELAVL3/4, BHLHE22, CALB1, CALB2, EBF3), ganglion cells (BRN3), and Müller glia (SOX2, RLBP1). These data confirm that this MRO system contains all major retinal cell types in a laminated structure (Völkner et al., 2016). In summary, MROs derived from the trisection protocol develop by regulated processes, so that stem cells become depleted and the generation of retinal neurons and glia is completed reproducibly. Thereafter, the MROs can be maintained in culture for further studies: Retinal cells remain postmitotic at least up to D60. Thus, it will be possible to use this system to study MRO maturation and stability at increasing organoid ages.

### Expression of Photoreceptor Markers and Phototransduction Machinery in MROs

**FIGURE 3:**
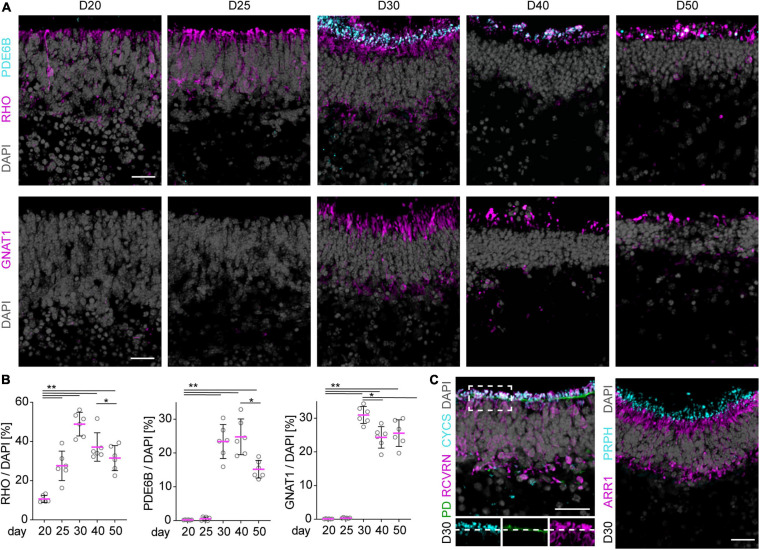
Photoreceptor maturation in MROs. **(A)** Representative images and **(B)** quantitative analysis of immunostained MRO cryosection. Rod specific marker RHO is already detected in a few cells in the ONL at day (D) 20 and becomes upregulated at D25. By D30, RHO is localized to the apical processes of the photoreceptors, and PDE6B as well as GNAT1 expression are detected in the same region. At later timepoints (D40, D50), all of these markers are still detected in the same region of the organoid, but the structures appear sparser and more disorganized. For quantification, pixel area above the threshold was measured for each marker and normalized to DAPI. Each dot represents an organoid, organoids were derived from *N* = 2 independent experiments. *p < 0.001, **p < 0.0001 (ANOVA). **(C)** Photoreceptors develop mitochondria-rich inner segments as indicated by localization of mitochondrial marker CYCS in apical RCVRN-positive processes above the outer limiting membrane highlighted by Phalloidin (PD) staining. The majority of the photoreceptors are rods (ARR1-positive). Localization of PRPH apical to ARR1 signal suggests the formation of outer segment like structures. Scale bars: 25 μm.

To characterize retinal cell maturation on a cellular level, we performed immunostaining analyses of known cell-specific proteins (markers) of the light receptive cellular structures and machinery. These become set up in newly-differentiated and maturing photoreceptors in the early postmitotic mouse retina [postnatal day (P) 5–10], respectively. Based on our data shown above and previously published, we estimated this stage corresponds to about stage D20–D25 and thereafter in our MRO system. Of note, retinogenesis in mice *in vivo* occurs in a central to peripheral wave, and is complete at about P5–P6 in the central retina, and at P10 in the periphery, and thus throughout the mouse retina *in vivo* (Young, 1985; Blackshaw et al., 2004; Ogawa et al., 2017; Brooks et al., 2019). We have not yet observed any evidence for an obvious central to peripheral wave in the MRO system based on our lineage-tracing and cell-proliferation studies (Figure 1, Völkner et al., 2016). Maturation is completed in the adult retina *in vivo* at P30 (Bonezzi et al., 2018): photoreceptors have a unique anatomical structure that is developed during maturation (Figure 2A); they are composed of a soma, and an outer (POS) and inner segment (PIS), which are located outside of the apical boundary of the retinal epithelium, also called the outer limiting membrane (OLM). The POS is the light-receptive structure of photoreceptors attached by a connecting cilium to the PIS, which contains mitochondria and the machinery required for lifelong POS renewal. The POS and PIS are formed during the maturation stage of postmitotic photoreceptors (Daum et al., 2017). In MROs at D20 and later, we observed that the outer retinal nuclear layer is composed of RHO, CRX+, and RCVRN+ photoreceptors (Figures 2B, 3A, and not shown). CRX regulates photoreceptor cell fate in development and function throughout life, whereas RCVRN controls the lifespan of rhodopsin (RHO), the light-sensitive receptor protein involved in visual phototransduction. Antibodies used for MRO immunostainings were validated on retinas derived from healthy mice at different postnatal stages (Supplementary Figure 3, not all shown). By staining for actin filaments (phalloidin, PD) and for mitochondria (CYCS), the OLM and PIS can be visualized in the retina and also in MROs (Figure 3C, D30). Based on marker expression studies for photoreceptors in postmitotic MROs (Figure 3C and Supplementary Figures 2B,D,E), the majority of photoreceptors in the MROs are rods (NR2E3+, ARR1+), and only a few are cones (ARR3+, OPN1SW+). RHO starts to be sporadically expressed at D20 within some scattered rods, and quantitative assessment shows that the number of RHO+ cells increases until D30 (Figures 3A,B). RHO becomes localized apically to the OLM at D25, and RHO+ stripe-like structures extend further in apical length at D30, indicative of PIS and POS formation and growth. However, this RHO staining pattern starts to appear less organized after D40. Other rod-specific proteins involved in phototransduction follow a similar expression pattern, but start slightly after the onset of RHO: Photon absorption by rhodopsin induces a signal transduction cascade in rod photoreceptors that leads to hydrolysis of cGMP by cGMP-phosphodiesterase (PDE), which leads to closed cGMP-gated channels and hyperpolarizes the cell. PDE6B is a part of PDE and becomes expressed at D30 (Figures 3A,B), colocalizing with the apical part of the RHO+ structures. Transducin alpha (GNAT1) stimulates the coupling of RHO with PDE, and its protein levels increase between P8 and P12 in mice *in vivo*, which coincides with the beginning of POS formation (Daum et al., 2017). In MROs, quantitative analysis of immunostained MRO sections showed an expression pattern of PDE6B and GNAT1 comparable to that found *in vivo*, starting with a very few cells at about D25 and strongly present at D30 (Figures 3A,B), indicating the beginning of POS biogenesis. GUCY2D is the photoreceptor guanylate cyclase that synthesizes cGMP and starts to be expressed at D30 (Supplementary Figure 2G). Proteins of the phototransduction machinery, including GNAT1, ARR1, GUCY2D, and RHO, localize or translocate from the ONL to outside of the OLM, indicative of PIS and POS formation and further maturation. Ultimately, latter mentioned proteins are maintained in the POS of mature photoreceptors; and photoreceptors without ARL13B, required for cilium formation, fail to develop a POS (Dilan et al., 2019). ARL13B and also peripherin (PRPH) are found at the apical boundary of the retinal epithelium in MROs (Figure 3C and Supplementary Figures 2C,F). In the maturing retina *in vivo*, ARL13B and PRPH localize to the distal tip of the connecting cilium of photoreceptors in advance of POS formation (Lee et al., 2006); PRPH is also essential for POS renewal. Cone photoreceptors are rather rare in the mouse retina (Völkner et al., 2016) and in MROs (Supplementary Figure 2D). Accordingly, the cone marker opsin blue (OPN1SW) could first be detected in a few cells of the outer layer of the MRO at D20 (Supplementary Figure 2E), although it outlined a clearer cone cell morphology at D30. Thus, age-dependent expression of photoreceptor-specific proteins indicates progressing retinal maturation comparable to mice *in vivo* (Figure 7A; Daum et al., 2017): Photoreceptor maturation is already occurring at D20, and various proteins of the phototransduction machinery seem to become expressed in a timed order. Immunostaining data indicate that PIS growth and POS formation possibly start and progress from D25 to D30 in MROs. To further assess this, we next performed ultrastructural analysis.

### Development of Inner and Outer Photoreceptor Segments in MROs

**FIGURE 4:**
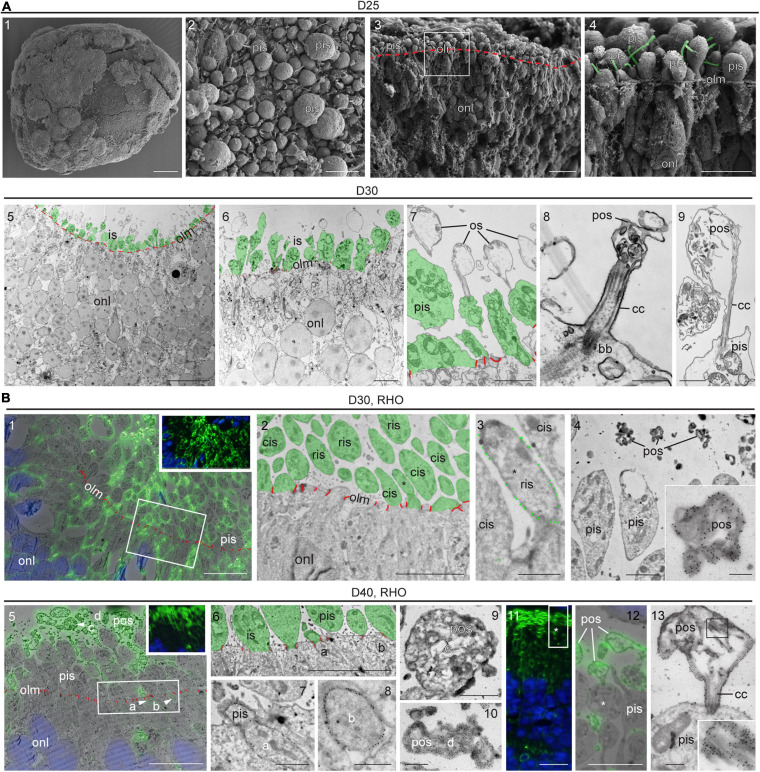
Ultrastructure of photoreceptor maturation and outer segments formation. **(A1–4)** Scanning electron microscopy (SEM) of D25 MROs. **(A1)** overview of whole organoid, **(A2)** surface of organoid with photoreceptor inner segments. **(A3,4)** SEM of retinal organoid after dissection, the sample fractured preferentially between cell borders (cross-section). **(A3)** Overview, the outer limiting membrane (i.e., apical surfaces of MRO cells) is indicated by the red dashed line. **(A4)** The region indicated by the square in **(A3)**. Cilia emerging from the inner segments are pseudo-colored in green. **(A5–9)** Transmission electron microscopy (TEM) of D30 epoxy embedded retinal organoid. Photoreceptor inner segments are pseudocolored in green and outer limiting membrane (cell junctions) is indicated in red. Inner segments are abundant **(A5,6)** and disorganized membrane structures (e.g., bubbles with disorganized membrane material) can be observed, that are linked to the photoreceptors by connecting cilia **(A7–9)**. These structures may represent outer segment equivalents. **(B)** Tokuyasu cryo-sections labeled with antibodies against rhodopsin (RHO) for correlative light electron microscopy (CLEM). **(B1–4)** D30, (**B1**: insert) immunofluorescence, (**B1**: overlay) fluorescence and TEM, note the membrane staining (inner segments and other parts of the photoreceptor cells). Non-labeled photoreceptor cells are presumably cones. The square indicates the region displayed in **(B2)**. **(B2)** Organoid surface with photoreceptors, RHO-positive inner segments are rod inner segments (ris), RHO-negative inner segments are cone inner segments (compare to **B1**), the asterisk indicates the inner segment shown in **(B3)**. **(B3)** Inner segments at higher magnification (2x cis, 1x ris/*), the gold particle labeling is highlighted by larger green dots. **(B4)** Inner segments and heavily stained photoreceptor outer segment equivalents (pos), one of which is shown at higher magnification (insert). **(B5–13)** D40 **(B5)** insert, immunofluorescence, **(B5)** overlay fluorescence and TEM. 4 ROI’s are indicated (arrowheads). The rectangle indicates the region shown in **(B6)**. **(B6–8)** surface of the MRO with two labeled parts of rods (ROI a and b), that are shown in **(B7,B8)** at higher magnification. **(B9,10)** Two photoreceptor outer segment equivalents (pos, ROIs c and d) at higher magnification. **(B11–13)** RHO-CLEM in a different region of the MRO. **(B11)** fluorescence, the rectangle indicates the region shown in **(B12)**, the asterisk indicates the ROI shown in B13. **(B12)** Overlay fluorescence and TEM, labeled pos are shown, one of which is connected to an inner segment (*). **(B13)** ROI with outer segment, connecting cilium (cc) and inner segment at higher magnification, insert shows heavily stained membrane structures of the outer segment from the region indicated by the square. D, day; olm, outer limiting membrane; pis, photoreceptor inner segment; ris, rod inner segment; cis, cone inner segment; pos, photoreceptor outer segment; onl, outer nuclear layer; cc, connecting cilium; bb, basal body; ROI, region of interest. Scale bars: **(A)** 1: 100 μm, 2: 5 μm, 3: 10 μm, 4: 5 μm, 5: 20 μm, 6: 5 μm, 7: 2 μm, 8: 500 nm, 9: 1 μm; **(B)** 1: 10 μm, 2: 5 μm, 3: 1 μm, 4: 2 μm, 4 insert: 500 nm, 5: 10 μm, 6: 5 μm, 7: 1 μm, 8: 500 nm, 9: 1 μm, 10: 500 nm; 11: 10 μm, 12: 5 μm, 13: 500 nm.

Some previous reports have suggested PIS and POS formation in MRO systems (Chen et al., 2016; Ito et al., 2017; DiStefano et al., 2018; Decembrini et al., 2020) but others have not (Eiraku et al., 2011; Gonzalez-Cordero et al., 2013; Hiler et al., 2015); not all studies have confirmed this using a gold-standard method yet. Further, it has been suggested that it might be necessary to experimentally stimulate POS formation (Busskamp et al., 2014; Brooks et al., 2019). To assess the formation of PISs and POSs in our MRO system we performed scanning electron microscopy of whole MROs before and after tissue fracture (SEM; Figure 4A1–4 and Supplementary Figure 4A), as well as using transmission (TEM; Figure 4A5–9 and Supplementary Figure 4B) and correlative (CLEM) electron microscopy of MRO sections (Figure 4B and Supplementary Figures 4C,D). PISs appear as protruding mushroom-shaped membrane structures that are infrequently found in MROs at D20 outside of the epithelium at the OLM (Supplementary Figure 4B1–2), but already occur in larger numbers at D25 (Figure 4A1–4 and Supplementary Figures 4A, 4B3–4). The characteristic mitochondria (Figure 4A7 and Supplementary Figure 4B5) and a large cilium (Figure 4A4,8) confirm that these structures are indeed PISs. Starting at D25, and very pronounced at D30, nascent POSs can be found at the tips of connecting cilia associated with PISs (Figure 4A6–9). This also matches the onset of GNAT1 expression, which becomes localized to the POS (Figure 3A). Outer and inner nuclear layers could be distinguished based on location, characteristics, and sizes of cell nuclei. Further, the OLM is characterized by cellular junctions between photoreceptor cells (Figure 4A and Supplementary Figure 4; red pseudocolored lines) and possible Müller glia processes at the apical organoid border. PISs and POSs can be definitely identified by electron microscopy, although it is not possible to discriminate between rods and cones at early maturation stages. Thus, the above-described anatomical structures were further confirmed by simultaneous immunofluorescence and immunogold labeling of recoverin (Supplementary Figure 4C) and RHO for CLEM (Figure 4B and Supplementary Figure 4D). These studies showed the cell connections between photoreceptors and Müller glia, which form the OLM (Supplementary Figure 4C), and RHO+ and RHO− PISs indicate rods and cones, respectively (Figure 4B2–3). Electron microscopy analysis confirmed early-developing POSs that were polarized to the apical side of the neural retina. POSs consisted of membrane evaginations at the tips of the connecting cilia that are tethered to PISs with a basal body (Figure 4A7–9). *In vivo*, they start to develop as loosely-organized, electron-dense membrane stacks that ultimately will contain the light-receptive machinery, and we find that these are indeed RHO+ (Figure 4B11–13). The developmental timing of POS formation at the structural level matched the expression observed for the photoreceptor markers described above, for example for ARL13B, a marker of ciliary axoneme (Figure 3 and Supplementary Figure 2). For further comparison, POS formation starts at P5 and rapidly progresses until P10 in mice *in vivo* (LaVail, 1973; Daum et al., 2017; Salinas et al., 2017). Thus, our data suggest that an MRO photoreceptor which matures at D25 is comparable to an *in vivo* retina at about P5–P8. Together, both the electron and light microscopic observations indicate rapid and progressive development of the specialized photoreceptor structures required for visual function as observed *in vivo* (Daum et al., 2017), and support progressing MRO maturation in longer-term culture.

### Formation of Synaptic Connections in MROs

**FIGURE 5:**
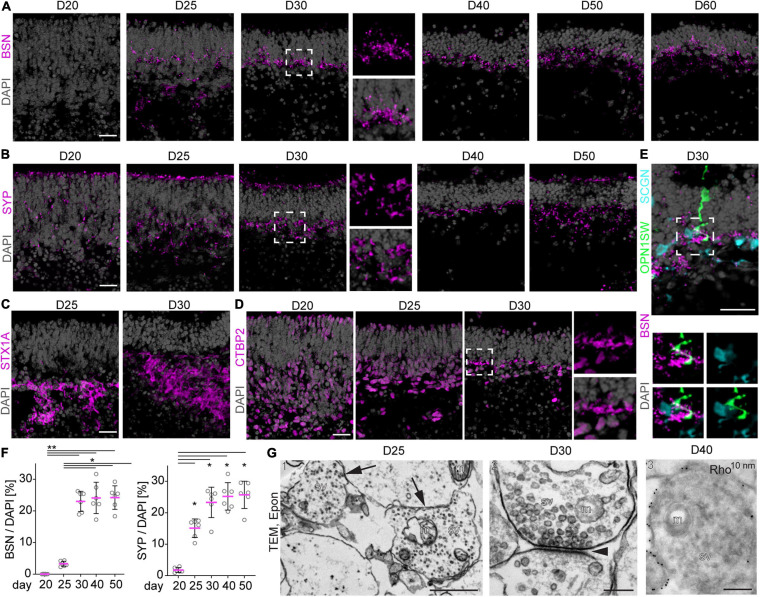
Synapse formation in MROs. **(A–E)** Representative images and **(F)** quantitative analysis of immunostained MRO cryosection at the timepoints indicated for synaptic markers BSN, SYP, STX1A, and CTBP2. Dashed squares indicate regions shown at high magnification. **(A)** BSN is first detected at D25, and becomes more prominent at D30 forming a dense outer plexiform (OPL)-like layer. At later timepoints, BSN is still detected in a dotted pattern in an OPL-like layer, but the expression becomes scarce. **(B)** Similarly, SYP is already expressed by a few cells from D20, but becomes localized to an OPL-like layer by D30. **(C)** STX1A is detected in more basal regions of the MRO at D25 and D30, but no clear inner plexiform like layer could be observed. **(D)** CTBP2 is first detected in nuclei of the majority of basal and a few apical cells at D20–25. At D30, small stretches of non-nuclear signal are observed in the OPL-like layer, suggesting formation of synapses. **(E)** At D30, synaptic marker BSN is detected in close proximity to the processes of cone marker OPNSW1- and cone-bipolar marker SCGN-positive cells, suggesting the formation of synaptic contacts between photoreceptors and second order neurons (bipolar cells). Dashed square indicate region shown at high magnification. **(F)** For quantification, pixel area above the threshold was measured for each marker and normalized to DAPI. Each dot represents an organoid, organoids were derived from *N* = 2 independent experiments. *p < 0.01, **p < 0.0001 (ANOVA). **(G)** Transmission electron microscopy (TEM) confirms the formation of synapses in D25 (1), D30 (2) and D40 (3) MROs. **(G1–2)** epoxy resin embedded samples, **(G3)** Tokuyasu cryo-sections stained with anti-RHO and protein A 10 nm gold. Arrows in **(G1)** indicate 2 synapses, arrowhead in **(G2)** indicates the synaptic cleft. m, mitochondrion; sv, synaptic vesicles; D, day. Scale bars: **(A–E)** 25 μm, **(G)** 1: 1 μm; 2, 3: 200 nm.

To assess another characteristic of retinal maturation, we determined whether neuronal synaptic connections are formed in MROs. The expression of well-established related proteins (von Kriegstein and Schmitz, 2003; Figures 5A–F) were studied by immunostaining MRO sections, and the ultrastructure was visualized by electron microscopy (Figure 5G). Synaptic connections in the vertebrate retina are organized into distinct laminae: the inner (IPL) and outer (OPL) plexiform layers between the three nuclear layers (Figure 2A). A network of axonal and dendritic processes with interconnected synapses of photoreceptor, bipolar, and horizontal neurons form the OPL, whereas the IPL consists of connections of bipolar, amacrine and ganglion cells. Bassoon (BSN), synaptophysin (SYP), syntaxin 1a (STX1A), and ribeye (CTBP2) in the mature mouse retina have presynaptic functions and label both synaptic layers during maturation in the developing mouse *in vivo* (Supplementary Figure 3). Ribbon synapses are characteristic structures located between photoreceptor and bipolar interneuron connections, the first- and second-order neurons in the retina. Bassoon and CTBP2 are two main components of the synaptic ribbon at the axon terminal. BSN is essential for photoreceptor ribbon synapse formation, CTBP2 is localized in the ribbon synaptic terminal of photoreceptors. Further, SYP is involved more widely in retinal synaptogenesis, and STX1A is believed to be involved in the docking of synaptic vesicles with the presynaptic membrane, and may play a role in outer and inner retinal formation. Our immunostaining analysis showed that BSN is still absent from the prospective synaptic layers at D20, whereas SYP started to appear, although still infrequently (Figures 5A,B). Five days later, BSN and SYP became strongly expressed in a pattern suggesting an OPL, and weakly indicating a potential IPL. Quantitative analysis of SYP and BSN immunostainings between D20 and D50 confirms increasing expression and thus maturation (Figure 5F). Further, STX1A (Figure 5C) clearly labeled a broad IPL and weakly labeled an OPL. CTBP2 became upregulated in the potential OPL at D30 (Figure 5D). Thus, OPL formation in MROs starts at D20 and progresses thereafter, which corresponds to P5–P7 in mice *in vivo* (Young, 1985). Synaptic proteins could be observed up to D60 in the OPL, and STX1A most clearly indicated the development and maintenance of a potential IPL. To further validate synapse formation, we performed co-staining of BSN with cell-specific markers for cones (OPNSW1) and a marker for a subpopulation of ON and OFF cone bipolar interneurons (SCGN, Supplementary Figure 2H). Colocalization between cell processes of OPN1SW+ cones and SCGN+ bipolars, together with BSN, indicates the formation of synaptic connections (Figure 5E). Electron microscopy of resin-embedded MROs at D25 and D30 confirmed synapses with synaptic vesicles, and the presence of synaptic mitochondria in several samples (Figures 5G1,G2 and Supplementary Figure 4E1). Immuno electron microscopy of cryo-sections with gold-labeled RHO antibodies also confirmed photoreceptor synapses (Figure 5G3Supplementary Figure 4E2; at D40). Together, changes in synaptic proteins indicate formation of an IPL and OPL starting at D20–D30, including synapse formation of photoreceptors with bipolar neurons. Further, changes in synaptic proteins indicate that synaptogenesis in the IPL and OPL is active during D20–D30. Plexiform layers in mice *in vivo* start to develop around P5, photoreceptor bipolar synapse start to form at about P7, and synaptogenesis is complete at about P21 (Akiba et al., 2019). Supporting our earlier studies showing that all retinal cell types are generated, the synapse data also suggest that although an IPL forms, the inner retina is not yet well developed at D20, and that this might not improve upon extended culture. However, our data also indicate that both plexiform layers are maintained up to D60. In summary, MRO extended culture reveals dynamic changes in synaptic proteins indicating the formation of synapses, an OPL, and an IPL. This further corroborates postmitotic maturation in this MRO system.

### Transcriptome Analysis of MRO Development and Maturation

**FIGURE 6:**
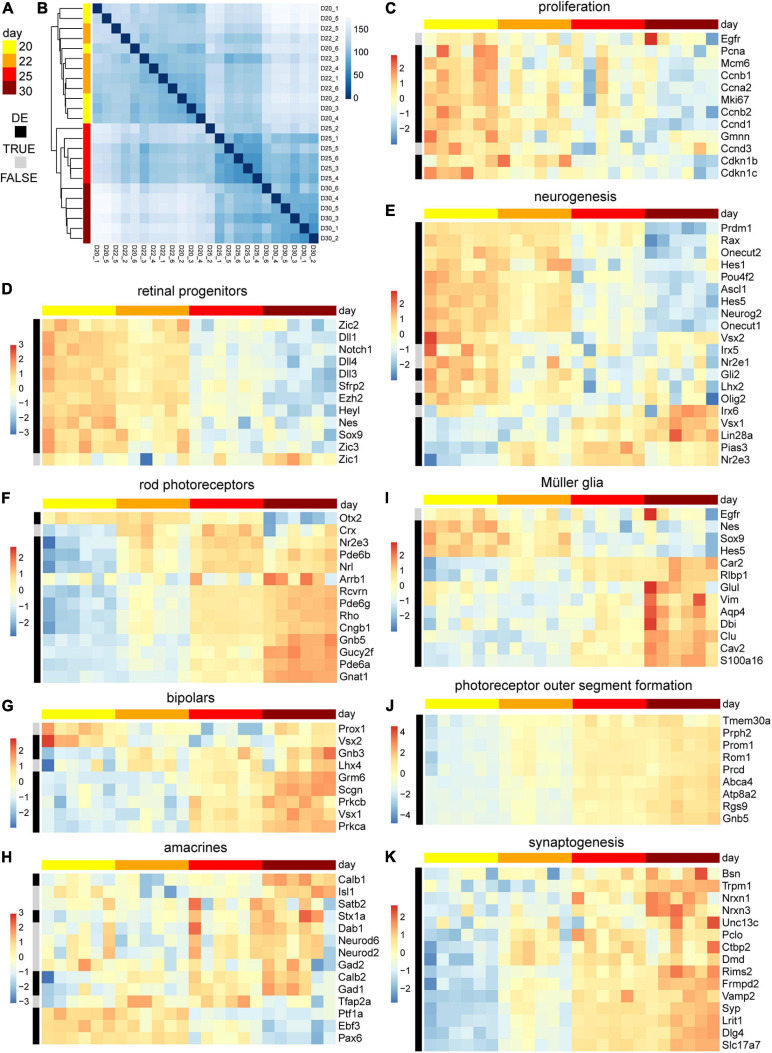
Transcriptome analysis of retinal maturation of the MRO system. **(A)** Color code of column and row annotation of the heatmaps indicate MRO sample age and differential gene expression (DE, FDR ≤ 0.01), respectively. D, day. **(B)** Sample-to-sample distance matrix (with clustering) for overall gene expression based on normalized counts shows low variation between individual samples per timepoint and a clear separation of the early (D20–22) and the later (D25–30) time points. **(C–K)** Heatmaps of selected genes of interest to study temporal changes in the gene expression associated with retinal progenitors, and neurogenesis of the major retinal cell types and their maturation. The heatmaps scales depict *z*-scores of log transformed counts. 6 MRO samples per timepoint were analyzed.

We sought to assess the molecular changes during the end of retinal cell birth and up to the onset of spontaneous pathology. Whole individual MROs were sampled at D20, 22, 25, and 30, and studied by RNA sequencing (6 MROs per timepoint). Reads were mapped to the genome and overlaps with annotated genes were counted. Sample-to-sample distances and principal component analysis of rlog transformed read counts show low variation between individual samples per timepoint (Figures 6A,B). The highest percentage of variance in the data, as explained by the first principal component with 59%, can be related to the developmental time of the samples (Supplementary Figure 5A). The differential gene expression analysis between the timepoints indicated a major change between D22 and D25 (Supplementary Figure 5B). Genes associated with cell proliferation, as well as with stemness and neurogenic competence, were more highly expressed in MROs at D20–D22, whereas their expression decreased at D22–D25 (Figures 6C–E): This includes genes like Pcna, Mcm6, Ccnb1, Cdkn1c, Egfr, and Vsx2, Notch1, Ascl1, Neurog2, Hes6, and Hes5, which are expressed in stem cells during retinogenesis *in vivo*, and become downregulated upon stem-cell depletion at P5 in the central retina and by P10 throughout the entire retina in mice (Blackshaw et al., 2004; Roesch et al., 2008; Nelson et al., 2011). Conversely, genes expressed in neuronal precursors and early postmitotic neurons (Figures 6D–H and Supplementary Figures 5C–E) become down- and upregulated around D20–D25, respectively: Nrl and Crx, which regulate and mark postmitotic photoreceptor precursors, peak from P4–P6 and P6–P8 in mice *in vivo*, respectively (Blackshaw et al., 2004), and both increase at least until D30 (Figure 6F). Interestingly, Nrl and Crx occur slightly later in another MRO system (Gonzalez-Cordero et al., 2013). To determine whether photoreceptors mature in MROs, we studied genes related to morphogenesis and phototransduction machinery, including those we studied by immunostaining (Figure 6F): Rcvrn, Rho, Gnat1, Pde6b, and others like Nrl, Nr2e3, increase between P6 and P12 in mice *in vivo*, at D22–D30 in our MRO system, and at D36 in others (Gonzalez-Cordero et al., 2013). Genes regulating POS formation (Rom1, Prcd, Prom1, and Prph2) are upregulated from D20 (Figure 6J), which is before we observed this at the ultrastructural level (Figures 3, 4). Related to the inner retina, transcription factors Vsx1 and Lhx4 are highly expressed in terminal differentiating and postmitotic bipolar cells at P5–P7 in mice *in vivo*, and at D22–D30 and D25–30 in MROs, respectively. Further, transcription factors Ptf1a and Tfap2a control commitment and specification of amacrines, respectively, whereas Neurod6 is expressed in postmitotic amacrines. These genes are enriched until and after D22, respectively (Figure 6H). Pou4f2 is an early and mature marker of retinal ganglion cells, and genes expressed in mature ganglion cells are detected from D22 (Supplementary Figure 5E, Pou4f2, Rbpms3, Thy1). Genes in mature amacrines (Tfap2, Rbfox3, Gad1, Calb1, Dab11) increase also from D22, and those in maturing bipolar cells (Grm6, Scgn, Prkcb) from D25 (Figures 6G,H), corresponding to the slightly later birth of bipolar cells during retinogenesis. Further, genes indicating synaptogenesis, like those (for example Syn, Bsn, and Stx1a) studied at the protein level (Figure 5) become enriched in MROs from D22 (Figure 6K). In addition, genes associated with Müller glia become upregulated in MROs from D22, including one expressed at postmitosis (Ttyh1), and those at a relatively high level upon maturation *in vivo* (Blackshaw et al., 2004; Roesch et al., 2008; Nelson et al., 2011), like Rlbp1, Dbi, Vim, Glul, Apoe, Aqp4, and Clu (Figure 6I). However, some genes indicative of mature inner retinal neurons (Pou4f2, Tfap2a, Pax6, Elavl3, Ebf3, Isl2) decline again at D30 (Supplementary Figure 5E), supporting the pathologic changes mentioned above. Bioconductors Ensemble of Gene Set Enrichment Analysis (EGSEA) was used to evaluate the overrepresentation of custom gene sets among the differentially expressed genes (FDR ≤ 0.01) in MROs over time (Supplementary Figure 6 and Supplementary Table 2). Genesets such as visual signal transduction of rods, visual perception, phototransduction, cilium, photoreceptor outer segment formation, and synapses, were upregulated, further supporting retinal development and maturation in the MRO system over time. In contrast, genesets such as Notch signaling, G2/M checkpoint of the cell cycle, and mRNA translation were downregulated, supporting the end of the retinogenesis. Taken together, this data shows a temporal gene expression pattern supporting stem-cell depletion and acquisition of a postmitotic stage in MROs at about D20–D22, a neuronal maturation starting and progressing from D20 to D30, and a subsequent development of potentially pathologic changes. Gene expression in MROs between D20 and D30 demonstrate a correlation with developmental stages, particularly those discriminating the end of cell differentiation and onset of maturation, as assessed by histology.

### Spontaneous Retinal Pathology Limits Maturation of MROs to Adult Stage and Their Maintenance

**FIGURE 7:**
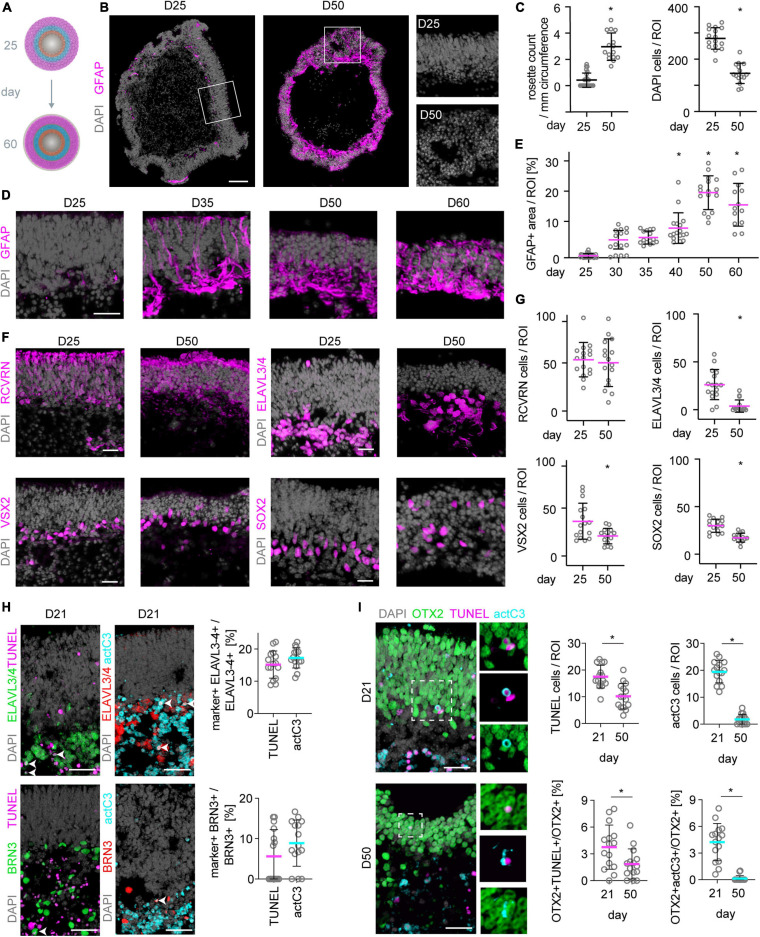
Neuronal cell death and reactive gliosis in postmitotic MROs. **(A)** Scheme experimental paradigm: MRO were assessed between day (D) 25 and 60 at the timepoints indicated in the figure. **(B,D)** Mouse retina organoid (MRO) cryosections were immunostained for gliosis hallmark GFAP and DAPI. At D25, no (or very little) GFAP is detected, but it becomes upregulated at later timepoints, suggesting the onset of reactive gliosis. Further, MROs at D25 show continuous long epithelia **(B)**, but at later timepoints (D50, **B**) epithelia become less organized and rosettes are frequently observed. Squares indicate regions shown at higher magnification. **(C,E)** Quantification of data presented in **(B,D)**. Each dot represents an organoid. **(F)** Staining for photoreceptor (RCVRN), inner neuron (ELAVL3/4), bipolar cell (VSX2) and Müller glia (SOX2) markers at D25 and D50 shows that inner neurons, bipolar cells and also Müller glia are reduced in numbers at later timepoints. In contrast, photoreceptor numbers remain similar at both timepoints. Notably also the total cell number (DAPI) decreases **(C)**. Further, the different cell types become displaced from their originally defined layers and more distributed, reflecting the epithelial disorganization and rosetting at later timepoints already noted above. **(G)** Quantification of data presented in **(F)**. **(H)** Representative images and quantification of immunostaining analysis for cell death markers activated caspase 3 (actC3) and TUNEL assay. BRN3 labels ganglion cells, OTX2 labels photoreceptors and bipolars. Legend: Each dot represents an organoid. *p < 0.01. D, day. Scale bars: **(B)** 100 μm, **(D,F,H,I)** 25 μm.

We show that MROs can be maintained longer in culture than previously reported. However, evidence also indicated pathologic changes. Thus, we investigated whether other pathology-related processes occur between D25 and D60 (Figure 7A): Microscopic analysis of whole MRO sections revealed changes in retinal epithelial structure, including epithelial folding and rosettes (Figures 7B,C), which are reminiscent of changes frequently observed in human patients with age-related macular degeneration and other retinal pathologies (Hariri et al., 2015). Next, we investigated whether Müller glia cells undergo reactive gliosis, a common and early response in most neurodegenerative conditions (Sardar Pasha et al., 2017). Transcriptome data (Supplementary Figures 5F,G) showed that genes associated with induction (Gfap, Cntf, Hbegf, Egfr) and regulation (Lif, Lifr, Mmp9) of reactive gliosis, and glia-derived endogenous neuroprotection (Bdnf, Cntf), are rather low or absent until D25, but increase thereafter. We confirmed upregulation of the reactive gliosis hallmark marker GFAP as a proxy: Immunostaining of MROs sampled at increasing ages between D25 and D60 showed that GFAP is mostly absent until D25 (Figures 7B,D,E). GFAP first increases slowly, becoming strongly upregulated at D40 (Figures 7D,E), subsequently to the onset of cell loss (Figure 1G). Quantitative analysis of retinal-cell composition at D25 compared to D50 (Figures 7F,G; *N* = 3 experiments, *n* = 5 MROs/N) showed that the number of RCVRN+ photoreceptors might be more variable but does not significantly decline (*p* = 0.68). However, cells in the inner retina become significantly reduced (Figures 7F,G): 85% of ELAVL3/4+ cells (amacrines, ganglion cells and horizontals; *p* < 0.0001), 43% of VSX2+ cells (subpopulation of bipolar cells; *p* = 0.0061), and 42% of SOX2+ cells (Müller glia; *p* < 0.0001) were lost. Previous reports of other MRO protocols also showed incomplete development and loss of inner retinal cells (Eiraku et al., 2011; Hiler et al., 2015; Ito et al., 2017; Brooks et al., 2019; Decembrini et al., 2020), but it is still unclear if developmental defects cause the outer retinal changes and overall deterioration.

Whether MRO stability and spontaneous pathology varies between different MRO protocols, is a key question in the field but beyond the scope of this work. However, in a preliminary experiment we tested a strategy that could be useful for future systematic comparisons of organoid systems. One experimenter performed parallel differentiation of MROs using two different protocols: Acutely dissociated mESCs were split into two fractions: One was used to generate MROs by the motherorganoid (Gonzalez-Cordero et al., 2013; Santos-Ferreira et al., 2016); the other followed the trisection protocol (Völkner et al., 2016). Analysis upon completion of retinogenesis (D23, Supplementary Figure 7) indicated a major difference in MRO stability: Motherorganoid MROs showed discontinuous retinal epithelia, and significantly increased and highly variable GFAP expression (*p* < 0.0001, *n* = 15 MRO each), indicative of reactive gliosis. In contrast, trisection MROs showed continuous epithelia with GFAP being rarely detectable (Supplementary Figure 7) confirming data above (Figures 7D,E). Thus, conceptual differences might not only determine MRO differentiation, yield and quality, but also longer-term stability. Since GFAP is not observed before D30 in our MRO system (Figures 4D,E), this supports the notion that pathologic cell death after end of retinogenesis might induce reactive gliosis. Of note, developmental cell death does not induce reactive gliosis *in vivo*, in contrast to wholemount culture of primary retinas (Supplementary Figure 8, Mervin and Stone, 2002; Löffler et al., 2015; Sardar Pasha et al., 2017). Given the potential different onset and phenotypes of outer (Ito et al., 2017) and inner (Brooks et al., 2019; Decembrini et al., 2020) pathologies in MROs, one question is whether this involves differential mechanisms, and cell death during or after retinogenesis.

### Exploration of Cell Death in the MRO System

We sought to study cell death in the MRO system to gain further insights into MRO stability and the onset of the spontaneous pathology. TUNEL assay and immunostaining for the apoptosis regulator active caspase 3 (actC3) were performed to assess cell death on MRO cryosections at D7, 10, 12, 15, 18, 21, 25, 28, 30 (*n* = 5 per timepoint, *N* = 1, Supplementary Figures 9A–C), and D21 in comparison to D50 (Figures 7H,I and Supplementary Figure 9D). Data suggested that cell death in MROs occurs in two waves (Supplementary Figures 9A–C), reminiscent of mice *in vivo* (Young, 1985; Chang et al., 1993; Portera-Cailliau et al., 1994; Laemle et al., 1999; Mervin and Stone, 2002; Péquignot et al., 2003; Doonan et al., 2007; Mao et al., 2008; Sancho-Pelluz et al., 2008): in the INL during late stage retinogenesis (P2–P10 *in vivo*; D18–D21 MRO); and in the ONL upon completion of retinogenesis (P5-15; D25-30). However, cell death was very rare after P18 *in vivo* (Young, 1985), whereas it was still occurring at D50 in MROs, mostly in the INL but also in the ONL (Figures 7H,I). To confirm this, we quantified cells colabelled with cell type and cell death markers at key timepoints: 15% of ELAVL3/4+ and 5% of BRN3+ cells underwent cell death at D21 (Figure 7H). Further, 4–5% of OTX2+ cells, a nuclear marker for photoreceptors and bipolar cells, underwent cell death at D21 (Figure 7I), whereas only 2% were TUNEL+ and almost none CASP3+ at D50. In summary, these data might explain the loss of inner retinal neurons in longer term culture, particularly amacrines and bipolar cells (Figures 7F,G), and provide a first insight into MRO developmental cell death. Although it is well established that a large number of newly-generated neurons do not survive due to programmed cell death in development *in vivo*, the mechanisms and exact cell numbers are still incompletely defined. Thus, a detailed comparison of MROs and *in vivo* retinas might decipher the mechanism of the ensuing pathology in MROs.

### Organoid Model for Enlarged Retina by Experimental Stem Cell Stimulation

**FIGURE 8:**
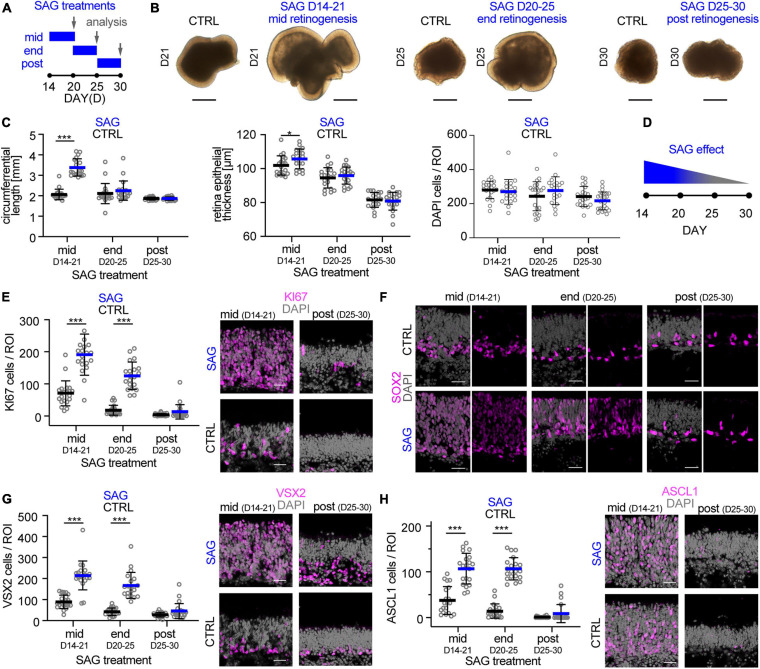
Experimental stimulation of cell proliferation for MRO enlargement. **(A)** Schematic of the experimental paradigm. Mouse retina organoids (MROs) were cultured with smoothened agonist SAG starting at mid (D14), end (D20) and post (D25) retinogenesis, and analyzed on D21, 25, and 30, respectively. **(B)** Representative phase contrast images of SAG treated and untreated (CTRL) MRO showing increase in organoid size at mid retinogenesis, but not at later timepoints. **(C)** Quantification of organoid circumference and epithelial thickness in entire MRO cryosections, as well as of total cells (DAPI) in regions of interest (ROI) of 100 μm width. Data confirms the increase of MRO size and epithelia thickness upon SAG treatment at mid retinogenesis, but not later. However, total cell counts per ROI do not increase, suggesting an expansion in epithelial length. **(D)** Summary schematic of the SAG induced effect on MRO. **(E–H)** Representative images and **(E,G,H)** quantification of KI67 (cell cycle marker), SOX2 (progenitors and Müller glia), ASCL1 (progenitors), and VSX2 (progenitors and bipolar cells) in untreated (CTRL) MROs and MRO treated with SAG. KI67 and SOX2 are upregulated in MRO treated with SAG at mid and end retinogenesis, but not post retinogenesis. The same is seen for ASCL1 and VSX2, however, VSX2 does also slightly increase in some SAG treated MRO at post retinogenesis. The potential of SAG to induce MRO growth declines rapidly with MRO age and maturation. *p < 0.05, ***p < 0.0001; D, day. Scale bars: **(B)** 500μm, **(E−H)** 25 μm.

**FIGURE 9:**
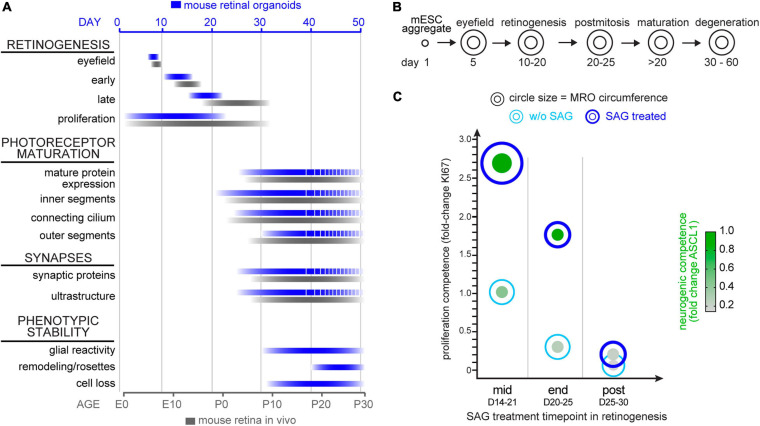
Summary schemes of MRO maturation and SAG-induced enlargement. **(A)** Summary of the development and maturation timeline of mouse retinal organoids (MRO, blue bars) as observed in this study and previously reported (Völkner et al., 2016, 2019) and in comparison, to *in vivo* mouse retina (black bars). Dotted lines indicate incomplete MRO maturation and pathologic changes. E, embryonic day; P, postnatal day. **(B)** Summary schematic of the phases of MRO development. mESC form an aggregate after 1 day (D) in culture and cells acquire eyefield identity starting from D5. From D10 to D20 all major cell types of the retina are generated in a defined order in MRO (retinogenesis). MRO become postmitotic and mature upon further culture, particularly between D20 and D30. From D30, pathologic changes occur in the inner and outer retina. **(C)** Summary of the SAG-induced effect on MRO at the different timepoints. Data shown are summarized from Figure 8: Circle diameter depicts MRO size based on organoid circumference. Proliferation competence depicted on the y-axis is based on KI67 data. Neurogenic competences presented as green dots is based on nuclear expression of ASCL1. Data presented were normalized to experiments started at mid retinogenesis (according to paradigms shown in Figure 8A).

The data above supports the notion that the trisection-based MRO system provides experimental access to at least four different developmental stages: (i) early and (ii) late retinogenesis; (iii) early postmitotic retinas (upon completion of retinal cell generation) when MROs are transiently stable in cell composition and continue to mature; and subsequently, (iv) retinas in extended culture undergoing pathologic changes. Previously, we have explored early retinogenesis and generated cone-enriched MROs generated by inducing premature differentiation (Völkner et al., 2016), and here sought to explore MRO age-dependent differences experimentally. We hypothesized that retinal epithelial length and thus organoid size could be reproducibly increased by controlled experimental manipulations, and with differential effects at different developmental stages. Controlling organoid size is of major interest, since a smaller or larger organoid may be an advantage or limitation depending on the research application, e.g., developmental studies, disease modeling or as an efficient cell source for single-cell applications, like preclinical cell transplantation studies. Increasing the number of starting mESCs does not result in increased MRO size, since MRO genesis becomes more variable and inefficient (Eiraku et al., 2011; Decembrini et al., 2014; Völkner et al., 2016). Thus, we proposed that experimental timed stimulation of retinal progenitor proliferation would increase MRO size. To test this, we used a potent sonic hedgehog (SHH) pathway activator, the chemical smoothened agonist (SAG) (Chen et al., 2002). Notably, activation of SHH signaling in LRP2-deficient mice *in vivo* causes a large eye phenotype (Christ et al., 2015). Further, it is well known that SHH contributes to retinal development, including retinal progenitor proliferation (Moshiri and Reh, 2004; Sigulinsky et al., 2008; Wallace, 2008; Wall et al., 2009; Christ et al., 2015), photoreceptor differentiation (Levine et al., 1997), patterning (Locker et al., 2006), and lamination (Wang et al., 2002). To determine the optimal timepoint for a transient stimulation, we applied SAG to MROs (Figure 8A) for several days either at mid- (D14), end- (D20), or post- (D25) retinogenesis (for details see methods). SAG applied in the middle of retinogenesis, but not later, reproducibly caused an elongation of the bright retinal epithelia in length that could be seen by microscopy live in culture (Figure 8B). Analysis on immunostained MRO sections confirmed this (Figures 8C,D): MROs significantly increased in size by 1.6-fold and with low variance based on the circumference of the central sections (*p* < 0.0001, CTRL: 2.1 mm COV 12.3%; SAG: 3.4 mm COV 12.5%, *N* = 4 experiments, *n* = 5 MROs/N), and epithelial thickness became slightly increased (*p* = 0.04, 102μm control, 106μm SAG). However, the total cell number based on DAPI+ nuclei count per retinal epithelia ROI remained unchanged (Figure 8C), indicating an expansion in epithelial length. Quantitative analysis of the cell-cycle marker KI67 and the mitotic marker phospho-histone-3 (PHH3) revealed that SAG significantly increased cell proliferation by 3- and 5-fold when SAG was applied in the middle (*p* < 0.0001) or at the end of retinogenesis (*p* < 0.0001), respectively, but not at postmitosis (*p* = 0.79) (Figure 8E and Supplementary Figure 10A). To find out if the proliferating cells are retinal progenitors, we assessed key transcription factors required for neurogenesis and retinal development. SOX2 regulates stemness in the retina, and remains expressed in Müller glia of the postmitotic retina. VSX2 regulates the multipotency of retinal progenitors and is expressed at the onset and throughout neurogenesis; its absence causes small retinas, and it remains expressed in a subpopulation of postmitotic bipolar neurons (Goodson et al., 2020). Further, ASCL1 is a pioneering factor driving neural development, required in postnatal retinal progenitors for neurogenesis, and is absent in the postmitotic retina (Brzezinski et al., 2011). In developing MROs, all three factors are expressed in the majority of cells in early retinogenesis, which declines with stem cell depletion over time (Figures 8F–H; Völkner et al., 2016). After the end of retinogenesis, SOX2 and VSX2 are expressed by Müller glia, and VSX2 also by a subpopulation of bipolar cells (Figures 2, 8F–H), whereas ASCL1 is absent. Qualitative assessment shows that SAG application increased SOX2 and VSX2 (Figures 8F,G), which previously were shown to mediate expansion of the pool of progenitor cells in the LRP2-deficient large eye model *in vivo* (Christ et al., 2015). Quantitative analysis (Figures 8G,H) confirms that VSX2+ and ASCL1+ cell numbers significantly increase by more than 2- to 3-fold each when applied either at mid (*p* < 0.0001 and *p* < 0.0001) or late (*p* < 0.0001 and *p* < 0.0001) retinogenesis compared to controls. In postmitotic MROs, ASCL1 remained absent upon SAG treatment like in controls, whereas VSX2 slightly increased (*p* = 0.4; Figures 8G,H). Relatedly, inhibitor of differentiation 1 (ID1) may stimulate stemness and SOX2 expression. The number of ID1 expressing cells increased when SAG was applied in the middle and at the end of retinogenesis, but not postmitotically (Supplementary Figure 10B). Together, this indicates that SAG might increase the number of progenitors and prevent differentiation. Further, the transcription factor OTX2 acts upstream of VSX2, high OTX2 levels facilitate neuronal differentiation, and VSX2 prevents cell differentiation by restricting OTX2 competence (Goodson et al., 2020). When SAG showed its strongest effect (mid-retinogenesis treatment), OTX2 became slightly reduced, but it remained unchanged when SAG was applied at the other stages (Supplementary Figure 10C), indicating the effects are dependent on the organoid age. Since immunostainings for photoreceptor (RCVRN) and Müller glia (SLC1A3) markers in enlarged MROs on D21 seemed comparable to controls (Supplementary Figure 10D), we wondered whether enlarged MROs still develop all the major retinal cell types in a stratified structure. To determine this, we analyzed MROs 5 days after the last SAG treatment (Supplementary Figure 10E): Immunostaining analysis showed that photoreceptors (RCVRN) were localized in the outer retina, whereas bipolar (PRKCA, VSX2) and amacrine cells (ELVAL3/4) and Müller glia (RLBP1, SOX8) were in the inner retina. Further, reactive gliosis was absent in SAG-enlarged MROs (like in controls) (Supplementary Figure 10F). Taken together, SAG stimulates MRO enlargement by increasing retinal progenitor proliferation, which becomes age-dependently restricted. Thus, the SAG effect might depend on the number of retinal progenitors still present at the time of stimulation. Application of SAG at D25, when retinal progenitors are already depleted, showed no effect on MRO size. However, some MRO cells at D20–25 but not D25–30, might still have the competence to re-enter the cell cycle, and upregulate stemness and neurogenic transcription factors. These data not only confirmed that MROs become postmitotic, but also that they remain postmitotic. In summary, these data support a proper completion of retinogenesis, and validate the reproduction of different developmental and maturation stages in this MRO system. Thus, the MRO system offers experimental access for studies at different stages (Figures 9A,B), and SAG treatment provides a protocol for the inducible generation of larger but still evenly-sized MROs (Figure 9C and Supplementary Figure 11).

## Discussion

Using our previously established trisection protocol optimized for reproducible generation of MROs, we assessed key parameters of MROs and their maturation in extended culture. MROs develop all major cell types in a layered structure, reach and maintain some key postmitotic characteristics, mature on a molecular and structural level, and can be maintained at least up to 50 days (Figure 9A). Our data indicate that this MRO system provides experimental access to embryonic, neonatal, and postmitotic retinas. However, maintaining MROs beyond 1 month is still limited, and it has not yet been possible to generate completely mature MROs with full retinal visual function, equivalent to the *in vivo* adult mouse. So far, this MRO system has been applied for studies of retinal development, establishing transgenic reporters, preclinical photoreceptor cell replacement therapy, and retinal cell biology (Völkner et al., 2016; Santos-Ferreira et al., 2019; Völkner et al., 2019). In addition to this, the presented data suggest that this MRO system might also facilitate studies of retinal maturation and the functions of newly-generated healthy and pathologic neurons and glia, for example retinal pathologies that commence during development or during retinal maturation in postmitotic retina, and including the spontaneous pathology as an experimental model. The reproducible developmental stages of this MRO system, the current deficiencies, and the spontaneous onset and dynamic progression of retinal neuropathologies and reactive gliosis provide insights and access for further optimizing the MRO system. These pathologic changes occur in MROs throughout stages that, based on the number of days in culture, might correspond to infant and adult stages *in vivo*. The potential applications and robustness of this MRO system at different developmental stages is confirmed by our experiments that led to age-dependent differentially-induced growth of larger-sized MROs (Figures 9B,C). The resulting modified protocol might be of interest for research projects requiring larger retinas or more retinal cells per organoid. Future studies will be required to determine if this phenotypic model for enlarged MROs still has the potential to acquire complete physiologic functions, involves additional pathologic changes, and reproduces a model for rare congenital enlargement of the eye, termed buphthalmos, in patients with Lrp2 mutations (Pober et al., 2009; Christ et al., 2015).

Previous studies established standardized assessment approaches and parameters at the cellular and molecular level: these can be used to perform reproducible quality controls for MRO generation (Gonzalez-Cordero et al., 2013; Decembrini et al., 2014; Hiler et al., 2015; Völkner et al., 2016; Brooks et al., 2019), and here we provide parameters for MROs in longer-term culture. As determined by our gene expression studies, cell birthdating, and progenitor and mitotic marker analysis of the MRO trisection protocol, all major retinal cell types have been generated and are present when retinogenesis is complete by about D20. Here, additional cellular and molecular studies confirm this, and we conclude that MROs homogenously complete retinogenesis, become postmitotic, and continue to mature over a transient timeframe. Notably, the acquisition of different MRO developmental stages was also supported by our SAG experiments that showed a strong increase in retinal progenitor cell proliferation during retinogenesis (D14), that this effect became age-dependently restricted (D20), and that it is absent at the postmitotic stage (D25). This indicates that progenitors become depleted, and that the retina not only becomes postmitotic but also maintains this state until D60. Previous studies have shown that retinogenesis finishes at between D20 and D35, depending on the MRO protocol: this indicates potential variances within and between MRO systems (Decembrini et al., 2014, 2020; Hiler et al., 2015; Chen et al., 2016; Völkner et al., 2016; DiStefano et al., 2018; Ueda et al., 2018; Brooks et al., 2019; Cui et al., 2020). The latter might be due to differences in the number of starting cells, and thus MRO size, or intraorganoid heterogeneity in retinal development. For example, each early neuroepithelium in organoids may develop more than one eyefield region, each of which may evaginate and give rise to a retina. If several such retinal domains develop within one organoid, these might compete with and disturb each other. Our trisection protocol reduces the size of the organoid prior to the onset of retinogenesis, which possibly reduces the development of large multi-domain retinal epithelia, such as has been observed in other protocols. Larger multi-domain MROs tend to convolute, which might limit their stability. Other major sources of variation are the pluripotent stem cell lines, experimenters, culture conditions, and variability in media supplements, particularly complex ones like Matrigel (Hiler et al., 2015; DiStefano et al., 2018; Slembrouck-Brec et al., 2018; Akhtar et al., 2019; Brooks et al., 2019; Capowski et al., 2019; Kaya et al., 2019; Mellough et al., 2019). So far, a systematic assessment of these parameters in MROs has been performed for different pluripotent stem cell lines (Hiler et al., 2015) and modified culture conditions (Chen et al., 2016; DiStefano et al., 2018; Brooks et al., 2019). Together, the parameters required for complete maturation and longer-term stability of mouse and also of human retinal organoids in culture are still incompletely defined, and the system and data presented here offer a basis to address this.

The cellular and molecular characterizations of several MRO systems have previously been reported during and at the end of retinogenesis, but not yet beyond this. Morphogenesis of photoreceptor cells, such as PISs, POSs, and ribbon synapses, and their connections to neighboring Müller glia forming the OLM, are essential for photoreceptor function. Studies in mouse retinas and MROs have shown that a major change in gene expression occurs at P6, indicating the switch from neurogenesis to postmitotic maturation (Daum et al., 2017; Brooks et al., 2019). In mice *in vivo*, POS formation starts at postnatal day P5 and rapidly progresses until end of retinogenesis (P10) and visual function starts with eye opening at about P12 (LaVail, 1973; Daum et al., 2017; Salinas et al., 2017). Our electron microscopy, immunostaining, and gene-expression studies support the development of several hallmarks of photoreceptor morphogenesis in extended MRO culture. PISs and POSs have previously been suggested and detected in some (Chen et al., 2016; Ito et al., 2017; DiStefano et al., 2018; Decembrini et al., 2020) although not yet in all MRO systems (Eiraku et al., 2011; Hiler et al., 2015), or only upon experimental stimulation (Busskamp et al., 2014; Brooks et al., 2019): not all studies confirmed this by the gold standard electron microscopy method. Notably, MROs with POSs showed light responses (Busskamp et al., 2014). Here, we show that photoreceptors and Müller glia form an OLM in MROs through cell connections at the apical retinal epithelial border. At this OLM, photoreceptors develop PISs and POSs. Thereby, we provided insight into the developmental time course: PISs start to be formed at D20, continue to grow, and develop characteristic mitochondria. Subsequently, PISs show connecting cilia with still rudimentary POSs at their tips, also called ciliary vesicles. At D30 they show a somewhat immature structure reminiscent of the disk formation characteristic of POSs. Further, we still detected POSs at D50, but these were not as extensively grown as *in vivo* ones. The observed changes in ultrastructure are also supported by timed expression of distinct genes and proteins, indicating photoreceptor maturation on a structural and functional level. In conclusion, our data show that PISs and POSs initially develop in MROs similarly to *in vivo* development, but certain signals might be missing for their ongoing growth and stabilization. Notably, MRO-derived whole-retinal epithelia or isolated photoreceptor cells show ongoing POS development upon transplantation into the mouse retina *in vivo* (Gonzalez-Cordero et al., 2013; Assawachananont et al., 2014; Decembrini et al., 2014; Lakowski et al., 2015; Santos-Ferreira et al., 2016). Thus, this postmitotic *in vivo* environment needs to be better mimicked in organoid culture. For example, MRO systems lack several cell types, like retinal pigment epithelium, as well as astrocytes, microglia, and vasculature that migrate into the retina during development (O’Sullivan et al., 2017; Silverman and Wong, 2018): these might be required for maturation and long-term maintenance of the physiological structure and function of the retina. Extracellular matrix components derived from the lens (Halfter et al., 2008; Oltean et al., 2016), other factors in the aqueous humor, and physical constraints like intraocular pressure, might also be important (Hosseini and Taber, 2018). Interestingly, supplementing with docosahexaenoic acid, a key component of POSs, facilitates MRO photoreceptor maturation, including PIS and POS formation (Brooks et al., 2019): whether this further improves long-term MRO stability has not yet been tested. Taken together, cellular, molecular, and ultrastructural data indicate that this MRO system completes retinogenesis at about D20–D25, since proliferation ceases (Figures 1, 6, and 8), and stem cells become depleted (Figures 7, 8; Völkner et al., 2016), which corresponds to about P5 in the central retina *in vivo*. Subsequently, MROs further mature from D25, with the formation of photoreceptor outer segments, both plexiform layers, and synapses being particularly notable, which occurs between P5 and P10 *in vivo*. For comparison, selected data in other MRO systems indicated that D26 in MROs corresponds to P4–P6 *in vivo* (Gonzalez-Cordero et al., 2013), D20 and D25 to P0 and P4–P6 (Decembrini et al., 2014), D28 to P6 (Hiler et al., 2015), D25–35 to P2–P6 (Chen et al., 2016), D23–D25 to P5–P7 (Ueda et al., 2018), and D26 to P6 (Brooks et al., 2019). Of note, modifying culture conditions may change the timing of MRO development: D25 in rotating vessel culture and D32 in static culture correspond to P6 (DiStefano et al., 2018). Here, our gene expression analysis indicates a major change between D22 and 25, which might be part of the previously described switch between retinogenesis and retinal maturation at P6 (Brooks et al., 2019). However, to resolve this a more systematic comparison of our MRO system and transcriptome data with previously-published data of other MRO systems and *in vivo* data is required to determine commonalities and differences compared to mice *in vivo* and to other MRO protocols, and to identify possible methods to facilitate the complete maturation and higher stability for long-term culture. Generally, our observations so far are in line with previous studies, indicating that retinal development and maturation are highly dynamic and partially comparable to mice *in vivo*.

Synaptic integration of neurons is a key process during retinal maturation and in gaining visual function. This integration is completed in the mouse *in vivo* at about P21 (Akiba et al., 2019). Here, we observed that synaptogenesis occurs in parallel to POS formation in the trisection-based MRO system, which is supported by temporal changes in synaptic-protein and gene expression. Further, our data show that progression of MRO maturation and the deficiencies involved can be studied in extended culture up to D60. Evidence for the development of synaptic layers and synapses in MROs was presented in the pioneering study (Eiraku et al., 2011). In subsequent work, this has been confirmed and extended at the cellular and molecular levels for some MRO systems (Gonzalez-Cordero et al., 2013; Hiler et al., 2015; Chen et al., 2016; DiStefano et al., 2018; Brooks et al., 2019; Capowski et al., 2019; Cui et al., 2020), but not yet for others (Decembrini et al., 2020). Notably, 3D electron microscopy of MROs has even shown well-formed synaptic ribbons in photoreceptor terminals (Hiler et al., 2015). Further, cell transplantation studies into the mouse retina *in vivo* also confirmed by electron microscopic and marker studies that MRO-derived donor photoreceptors have the plasticity to develop synaptic connections with host inner nuclear neurons (Gonzalez-Cordero et al., 2013; Assawachananont et al., 2014; Decembrini et al., 2014; Santos-Ferreira et al., 2016). However, although synapse formation occurs in MROs, deficits of the inner retina have been observed (Decembrini et al., 2014; Völkner et al., 2016; DiStefano et al., 2018; Brooks et al., 2019). Here, we find that this is also supported by incomplete expression of synaptic markers in the IPL, and although plexiform layers are maintained, this is not resolved in extended culture. Previous studies have shown that the primary cell source might be a cause for this, since pluripotent stem cells with a retinal epigenetic memory, like those derived from retinal cells by reprogramming, improve inner retinal development (Hiler et al., 2015). However, recent adaptations of an MRO protocol that doesn’t form synapses in classic (floating) organoid culture conditions, have shown that rotating culture conditions improve inner retina and synapse development, even though the originating pluripotent stem cells were not retinal derived (DiStefano et al., 2018; Wang et al., 2018). FGF1-treated MROs also showed improvements in synaptic-marker expression (Brooks et al., 2019), whereas removing serum shortened survival (Ito et al., 2017). Further, O_2_ tension in MROs might need to be optimized for gas exchange (Chen et al., 2016). O_2_ might become limited with increasing epithelial thickness. Whether increased size impairs the quality and progress in maturation and synapse formation still needs to be determined. Defined organoid protocols offer the advantage of being tailored for specific application requirements, and provide a simplified system compared to the *in vivo* counterpart. Although there is a clear need and interest to integrate additional components in the future, a simplified system requires fewer quality controls and increasing organoid complexity might also limit or even reduce MRO stability. So far it remains unclear if any additional components might be required to achieve complete MRO maturation and long-term maintenance.

The major limitations of, and differences between, different retinal organoid protocols, using mouse or human pluripotent stem cells, are still unsolved, particularly for longer-term studies. The pioneering study by Eiraku et al. (2011) already described that retinal integrity in MROs decreases after D35, and subsequent studies have reported variable onset of inner retinal deficits, and deterioration between D20 and D30 (Hiler et al., 2015; Chen et al., 2016; Ito et al., 2017; DiStefano et al., 2018; Ueda et al., 2018; Brooks et al., 2019; Decembrini et al., 2020). Notably, in the trisection system presented here, the neurons of the inner retina decrease and reactive gliosis started about D30, and although rosette formation occurred in the outer retina the photoreceptor cell number remained largely unchanged up to D50. This possibly provides a window of opportunity to experimentally model induced photoreceptor and glial pathologies in MROs. Within this time window, the only study so far of retinal pathology modeling in MROs reported experimentally-inducible photoreceptor cell death between D23 and D27 (Ito et al., 2017). However, MROs under control conditions already showed inner retinal defects prior to the onset of the experiment, and maintained outer retinal morphology only up to D30. Thereafter, induced photoreceptor cell death could not be distinguished from spontaneous degeneration in controls: this limited longer-term studies of photoreceptor pathology. Whether such differences between organoid protocols are due to different cell-culture conditions, experimenters, or cell lines is still unclear. Here we show that MROs in the motherorganoid protocol already showed pathologic reactive gliosis right at the end of retinogenesis (D23), whereas MROs in the trisection system develop this about 1 week later. In order to further address such questions in the future, in these preliminary studies we assessed a strategy to systematically compare protocols by parallel differentiation.

Organoids are tunable systems that can be adapted to experimental needs. However, increasing the amount of a certain cell type may require compromises, like a decrease in other cell types or a higher variance. For example, premature induction of retinal differentiation in MROs results in a high number of cone photoreceptors at the cost of rods and overall structure (Völkner et al., 2016). And a higher variance might require additional MRO dissection to exclude any variable contribution of non-retinal parts just prior to the experiment (Ito et al., 2017). Here, we show that SAG-stimulated MROs were reproducibly larger. This modification of the trisection protocol combines two advantages into one system: a high yield of similarly-sized MROs; and a longer retinal epithelium. Various experimental applications might benefit from this. For example, larger MROs provide more sequential tissue sections and cells per organoid whenever single organoids are needed for intra- and interorganoid comparative studies. Also, larger MROs provide more cells for single-cell applications, like cell transplantation studies, and reduce the number of separate differentiation batches for larger-scale experiments. It will be interesting to learn if SAG treatment works similarly in other MRO systems, in human organoids, and primary retina *in vivo*. Larger organoids have also been developed in other neural organoid systems, and introducing biomaterials as a growth substrate has optimized differentiation and stabilization (Lancaster et al., 2018). At a certain size, this might also be required for retinal organoids, which may become convoluted and therefore deteriorate. Generally, MROs are larger in other protocols, with an estimated surface area ranging between 2.5 and 7 mm^2^ (Decembrini et al., 2014; Völkner et al., 2016; DiStefano et al., 2018). For comparison, we estimated the surface area of MROs based on their circumference by assuming that the organoid is spherical. At D25, in our MRO system it is 1.4 mm^2^, which is about one-tenth of a primary mouse retina at the end of retinogenesis (∼14 mm^2^ P10 flatmount, Völkner et al., 2016), and SAG treatment increased it by 2.6-fold (to 3.7 mm^2^). Although many SAG-stimulated MROs showed seemingly homogenous expansion in retinal epithelial length, some showed multiple differentially expanding retinal epithelial domains: this resulted in multi-lobated structures. It is still unclear if MROs can be generated from one eyefield-like patch of cells of origin, or how many separate retinal epithelia develop together on average within each MRO. One explanation might be that SAG-induced epithelial expansion might reveal this, which would be why some show multi-lobated structures. Alternatively, SHH-meditated signaling has multiple functions in retinal development, and might differentially induce progenitor expansion and differentiation. So far, our data indicate that SAG-derived MROs contain all major cell types in a layered structure, but the cell ratio might be different. This requires birthdating and more quantitative studies. Generally, it is well known that retinas may grow by different mechanisms. At least in some species, retinal size is determined by an expanding wave of progenitors, and their differentiation involves SHH signaling (Masai et al., 2000; Neumann and Nuesslein-Volhard, 2000). Retinal ganglion cells are early-developing neurons in vertebrate retinas that express SHH, which promotes retinal progenitor proliferation and differentiation toward neuronal cell types (Esteve and Bovolenta, 2006). Further, retinas in some animals grow by the addition of new neurons from a growth zone at the retinal margin (Moshiri et al., 2004; Tsingos et al., 2019). Although this region is not involved in mouse and human development, it is part of their retinas (Bhatia et al., 2009): progenitor expansion can be experimentally stimulated and involves SHH signaling (Moshiri and Reh, 2004; Moshiri et al., 2004, 2005; Bhatia et al., 2009; Christ et al., 2015). In human patients, increased eye size (buphthalmos) may be caused by mutations in SHH signaling pathways (Pober et al., 2009). Our data indicate that all major cells types are generated in SAG-enlarged MRO, but it is not clear whether increased epithelial length might be the only pathologic change and if other aspects of buphthalmos are reproduced. SAG stimulation of MROs during development may either occur by increasing the number of progenitors throughout the retina and thereby increasing the number of all or some retinal cell types; or specifically increasing the number of differentiating photoreceptors. Further, SAG might expand retinal epithelia by expanding progenitors in margin-like areas and thereby by appending epithelial growth. However, the observed increase in ASCL1+ cells throughout the retinal epithelia supports overall progenitor expansion, which argues against a margin-like mechanism. Further, manipulation of SHH signaling has previously been shown to induce or extend progenitor proliferation in the retinal margin after the end of retinal development. In our MRO studies it is not yet clear whether SAG at mid and end of retinogenesis increases the number of proliferating cells by stimulation of the cycling stem cells, by inducing cell-cycle re-entry of postmitotic cells, or both. However, SAG had no effect in postmitotic (D25) MROs, which might also argue against a margin-like mechanism, and this also suggests that SAG is not sufficient to induce Müller glia proliferation at this point in healthy, postmitotic MROs. Studies in chick and mouse retinas have shown that SHH can stimulate Müller glia proliferation and Müller glia derived neuronal regeneration in damaged but not healthy retina (Wan et al., 2007; Todd and Fischer, 2015; Kaur et al., 2018; Thomas et al., 2018). Further, glial proliferation has been shown to become age-dependently limited in the early postmitotic retina (Löffler et al., 2015), and SHH-dependently in other parts of the nervous system (Heimann et al., 2017). Thus, here we probed the stem-cell competence of embryonic and postmitotic MROs by applying SAG at increasing ages: this provided an insight into its proliferative competences and maturation. In summary, the established SAG-stimulated MRO system facilitates retinal enlargement, and shows that MROs offer an efficient assay to study retinal growth and extrinsic manipulations.

## Data Availability Statement

The datasets presented in this study can be found in online repositories. The names of the repository/repositories and accession number(s) can be found below: NCBI GEO (accession: GSE168139).

## Ethics Statement

The animal study was reviewed and approved by Landesdirektion Dresden, 09105 Chemnitz, Germany. Written informed consent was obtained from the owners for the participation of their animals in this study.

## Author Contributions

MV and MK contributed to conceptualization and writing. MV performed the MRO experiments. MV, LE, JS, LB, CK, JH, and MK analyzed the data. TK performed the electron microscopy imaging. TK, MV, and MK analyzed the data. All authors contributed to the article and approved the submitted version.

## Conflict of Interest

The authors declare that the research was conducted in the absence of any commercial or financial relationships that could be construed as a potential conflict of interest.

## Footnotes

1 http://www.janvier-labs.com
