# Supplementary material for: Mouse Retinal Organoid Growth and Maintenance in Longer-Term Culture
Source: Front Cell Dev Biol. 2021 Apr 27;9:645704. doi: 10.3389/fcell.2021.645704 (PMC8114082; doi:10.3389/fcell.2021.645704)
Supplement: Supplementary file 3 [file Data_Sheet_1.docx]

***Supplementary Material***

**Supp. Fig.1:** Extended characterization of retinal cell types in MROs.

Representative images of immunostained cryosections of (A) MROs at D20 compared to (B) primary mouse retina at postnatal day 6. Marker label bipolars and amacrines (PROX1, BHLHE22), or amacrines (EBF3, CALB1, CALB2). Scale bars: 25µm

**Supp. Fig.2:** Extended analysis of MRO maturation.

(A) Pan photoreceptor marker CRX is expressed in the complete outer nuclear layer (ONL) at D20, D25 and D30. (B) Rod specific marker NR2E3 is expressed in the majority of the cells in the ONL. (C) ARR1 is expressed already at D25, but PRPH2 is only detected at D30 (see Fig.3). (D) Cone photoreceptor marker ARR3 is only rarely detected in mouse retinal organoids (MRO), suggesting a rod-dominated phenotype. (E) Cone marker opsin blue (OPN1SW) could be detected in a few cells already at D20 and a clearer cone cell morphology could be observed by D30. (F) ARL3B is located apically to the OLM, indicated by Phalloidin (PD) staining. (G) Mature photoreceptor marker GUCY2D is expressed in the apical processes of photoreceptors at D30, but not before, suggesting photoreceptor maturation in MRO. (H) Bipolar cell marker SCGN is detected in the inner organoid at D25 and D30. D – day. Scale bars: 25µm (all images).

**Supp. Fig.3:** Photoreceptor and synaptic markers defined in the mouse animal-derived retina.

Mouse retinal cryosections were immunostained for photoreceptor maturation markers (A) ARR1, (B) RHO, (C) GNAT1, (D) PDE6B and (E) GUCY2D, as well as synaptic markers (F) CTBP2, (G) BSN and (H) SYP. Photoreceptors show an organized and gradual maturation process in the postnatal mouse retina. RHO is first detected in a few cells of the outer retina at postnatal day (p) 0 (birth), and becomes upregulated and localized apically to the outer segments by postnatal day (p) 6. ARR1 and PDE6B are detected from p6, while GNAT1 and GUCY2D are only observed at p11. Similarly, also synaptic markers show a gradual onset. SYP is already detected at p0 in the developing inner plexiform layer and becomes more strongly expressed by p6. At p11 it is also detected in the outer plexiform layer. In contrast, BSN and CTBP2 are not expressed at p0, but only at p6 in the inner plexiform layer. At p11, both are upregulated and also detected in the outer plexiform layer. p – postnatal day. Scale bars: 25µm.

**Supp. Fig.4:** Extended data on photoreceptor maturation at the ultrastructural level.

(A) Scanning electron microscopy (SEM) of a dissected D25 organoid. The sample fractured preferentially between cell borders, cross-section. (A1) overview, the outer limiting membrane (i.e. apical surfaces of organoid cells) is indicated by red dashed lines. (A2) region indicated by square in A1. Cilia emerging from the photoreceptor inner segments (pis) are pseudo-colored in green. (B) Transmission electron microscopy (TEM) analysis of epoxy resin embedded day 20 and 25 MROs. Photoreceptor inner segments are pseudocolored in green and the outer limiting membrane is indicated by the red dashed line (B1-4). Well-developed inner segments are rare at D20 (B1,2), but quite abundant at D25 (B3,4), and sometimes carry a connecting cilium (B5). (C, D) Tokuyasu cryo-sections of MROs labeled with antibodies against RCVRN (C) or RHO (D) for TEM (C) or CLEM (D). (C1-3) D30, RCVRN immunogold labeling. Overview (1) and details (2,3). All photoreceptors are labeled with 10 nm gold particles indicated by larger brown dots (C2,3, immunogold labeling: anti-RCVRN, ProtA 10 nm gold). Section planes are perpendicular (2) or approximately parallel (3) to the organoid surface. Immunogold labeled photoreceptors or inner segments (pis, in C1) are pseudocolored in green, unlabeled Müller glia in blue. (D1-7) D50, RHO immuno-CLEM (D1) fluorescence overview, the region indicated by the square is shown in D2. (D2) overlay fluorescence and TEM at organoid surface; note the reduced membrane staining; 2 ROIs are indicated (arrowheads). (D3) ROI 1 is one of the rare photoreceptor inner segments (pis) with membrane staining, (D4) ROI2 is a heavily labeled photoreceptor outer segment (pos). (D5-7) subsurface outer nuclear layer. (D5) insert fluorescence, a single membrane labeled photoreceptor (nuclear level) is indicated (*), (D5) overlay fluorescence and TEM, ROI indicated (*). (D6) photoreceptor with membrane staining (gold particles highlighted by larger green dots). The square indicates the region shown in D7. (D7) Membrane staining at higher magnification (Immuno fluorescence and gold labeling D41-7: anti-RHO, Goat-anti-rabbit Alexa488, Prot A 10 nm gold). (E) Transmission electron microscopy (TEM) confirms the formation of synapses in D30 (1) and D40 (2) MROs. (E1) epoxy resin embedded sample, (E2) Tokuyasu cryo-section stained with anti-RHO and protein A 10 nm gold. Legend: D – day, olm – outer limiting membrane, pis – photoreceptor, pos – photoreceptor outer segment, onl – outer nuclear layer, cc – connecting cilium, mg – Müller glia, m – mitochondrion, sv – synaptic vesicles, ROI – region of interest. Scale bars: (A) 1: 5µm, 2: 2µm; (B) 1,3: 20µm, 2: 2µm, 4: 5µm, 5: 1µm; (C) 1: 10µm, 2: 1µm, 3: 500nm; (D) 1: 50µm, 2,5: 10µm, 3: 1µm, 4,7: 500nm, 6: 2µm; (E) 1, 2: 500nm.

**Supp. Fig.5:** Supplemental data for transcriptome analysis of the MRO system.

(A) Principal component (PC) analysis (PCA) of the rlog transformed normalized count data. (B) Heatmap plot depicting differentially expressed genes for all timepoints (FDR 0.01). (C) Color code for heatmap plots indicating MRO sample age and statistical significance of differentially expressed genes (DE). Heatmaps scales depict z-scores of rlog normalized counts. 6 MRO per timepoint were analyzed. (D-E) Selected genes of interest to study temporal changes in gene expression associated with major retinal cell types and (F-G) reactive gliosis and neuroprotection response in retinal pathologies.

**Supp. Fig.6:** Ensemble of Gene Set Enrichment Analysis of MRO development.

Ensemble of Gene Set Enrichment Analysis (EGSEA): Analysis was performed for MRO at day (D) 20, 22, 25 and 30. Analysis based on different EGSEA databases (A – GeneSetDB Gene Ontology (GO), B – h Hallmark Signatures, C – c5 GO Gene Sets, D – GeneSetDB Pathway) revealed several significantly enriched terms supporting temporally regulated retinal development and maturation of the MRO system. The z score states how many standard deviations from the population mean a given score is. Detailed data for each plot is provided in Supplementary Table 2.

**Supp. Fig.7:** Comparison of retina organoid stability in the motherorganoid versus trisection protocols.

(A) Mouse retinal organoids (MRO) were differentiated in parallel from the same batch of mESC using the trisection (TRIP, (Völkner et al., 2016; Völkner et al., 2019)) and motherorganoid (MOP, (Gonzalez-Cordero et al., 2013)) protocols, respectively. See methods for details on the protocols. (B) Tissue sections of MROs at day 23 were immunostained for photoreceptor (RCVRN), reactive gliosis (GFAP), and cell nuclei (DAPI) markers. MROs derived from the trisection protocol showed continuous epithelia with photoreceptors localized in an outer nuclear layer-like structure along the outer (apical) surface of the organoid, as well as low (or no) GFAP expression. In contrast, MROs derived from the motherorganoid protocol showed increased and highly variable GFAP expression, as well as discontinuous retinal epithelia. (C) Quantification of GFAP expression. Each circle represents one individual organoid (n=15) derived from one independent differentiation round. Error bars represent standard deviations (SD). * p<0.0001 (Student’s t-test). Scale bars: 200µm.

**Supp. Fig.8:** Absence of reactive gliosis in primary retina in vivo and spontaneous induction in culture.

(A) Mouse retina was immunostained for Müller glia marker RLBP1, reactive gliosis hallmark GFAP and DAPI. RLBP1 positive Müller glia radially span the entire width of the postnatal day (p) 6, 11 and 35 mouse retinas. GFAP expression is not observed in the radial Müller glia, but only in astrocytes residing in the ganglion cell layer, confirming that in situ Müller glia do not become reactive. (B) In contrast, if p10 mouse retina is ex vivo cultured for 4 days (p10: DEV4) (see methods for details on culture protocol), GFAP is upregulated in the radial Müller glia, suggesting that they become gliotic upon ex vivo culture. (C) Quantification of GFAP expression in the mouse retina at p6, p11 and p35, as well as in p10 ex vivo cultured mouse retina at DEV4 and DEV6. Each dot represents one retina. * p<0.0001 (ANOVA). p – postnatal day, DEV – day ex vivo. Scale bar: 25µm.

**Supp. Fig.9:** Cell death analysis in the MRO system.

(A) Representative images and (B-C) quantitative analysis of cell death in the MRO system based on TUNEL assay and immunostaining of activated caspase 3 (actC3) on MRO cryosections at several selected timepoints. Neurogenesis was monitored in parallel based on ELAVL3/4 (marker for immature and mature amacrines, horizontals, ganglion cells), which matched the birthdating we observed previously (Völkner et al. 2016). (B) Analysis was performed per retinal layer, outer (ONL) and combined inner nuclear layer and ganglion cell layer (INL/GCL); indicated by white dashed lines in (A). Based on marker location, cell death possibly peaks in ganglion and amacrine cells at D18-21, and bipolars and photoreceptors at D25-28, which occurs at P2-5 and P7-8 in vivo, respectively. (D) Data related to Fig.7I: OTX2 labels photoreceptors and bipolars, and DAPI all cell nuclei. Each dot represents one organoid (n), (B-C) n=5, 1 independent experiment (N), (D) n=5/N, N=3. * p<0.05, ** p<0.01, *** p<0.0001 (ANOVA). Scale bars: 20µm

**Supp. Fig.10:** Extended data on experimental stimulation of retinogenesis.

Related to Fig.8. (A-C, F) Representative images and quantification of PHH3 (mitosis marker), ID1 (progenitors), OTX2 (progenitors, bipolars and photoreceptors) and GFAP (reactive gliosis marker) in untreated (CTRL) MROs and MRO treated with SAG. (D) Mouse retina organoids (MRO) treated with or without (CTRL) smoothened agonist SAG to stimulate retinal growth, were immunostained for photoreceptor marker RCVRN and Müller glia marker SLC1A3, and DAPI (grey). MROs treated with SAG at mid retinogenesis express lower levels of RCVRN compared to CTRL, suggesting a less mature state. However, MROs treated at late retinogenesis time point show similar RCVRN levels as CTRL organoids. Both MROs treated at mid and late retinogenesis express Müller glia marker SLC1A3 at similar levels as CTRL organoids, suggesting no effect of SAG treatment on Müller glia development.

(E) Representative images of MROs analyzed five days after (on day 26) the last SAG treatment from day 14 to 21: photoreceptors (RCVRN) are localized in the outer retina, whereas bipolars (PRKCA, VSX2), amacrines (ELVAL3/4) and Müller glia (RLBP1, SOX8) are in the inner retina. Scale bars: 25µm.

**Supp. Fig.11: Graphical abstract.**

**Supp. Tab.1:** List of antibodies used in this study.

Cat.No.- Catalogue number. CLEM – correlative light and electron microscopy

**Supp. Tab.2:** EGSEA data related to Fig.S6.

GO – Gene Ontology, MF – Molecular Function, BP – Biological Process, CC – Cellular Component.

**Supplemental References**

Gonzalez-Cordero, A., West, E.L., Pearson, R.A., Duran, Y., Carvalho, L.S., Chu, C.J., et al. (2013). Photoreceptor precursors derived from three-dimensional embryonic stem cell cultures integrate and mature within adult degenerate retina. *Nat Biotechnol* 31(8)**,** 741-747. doi: 10.1038/nbt.2643.

Völkner, M., Kurth, T., and Karl, M.O. (2019). The Mouse Retinal Organoid Trisection Recipe: Efficient Generation of 3D Retinal Tissue from Mouse Embryonic Stem Cells. *Methods Mol Biol* 1834**,** 119-141. doi: 10.1007/978-1-4939-8669-9_9.

Völkner, M., Zschatzsch, M., Rostovskaya, M., Overall, R.W., Busskamp, V., Anastassiadis, K., et al. (2016). Retinal Organoids from Pluripotent Stem Cells Efficiently Recapitulate Retinogenesis. *Stem Cell Reports* 6(4)**,** 525-538. doi: 10.1016/j.stemcr.2016.03.001.
