# Supplementary figures and images for: Mouse Retinal Organoid Growth and Maintenance in Longer-Term Culture
Source: Front Cell Dev Biol. 2021 Apr 27;9:645704. doi: 10.3389/fcell.2021.645704 (PMC8114082; doi:10.3389/fcell.2021.645704)

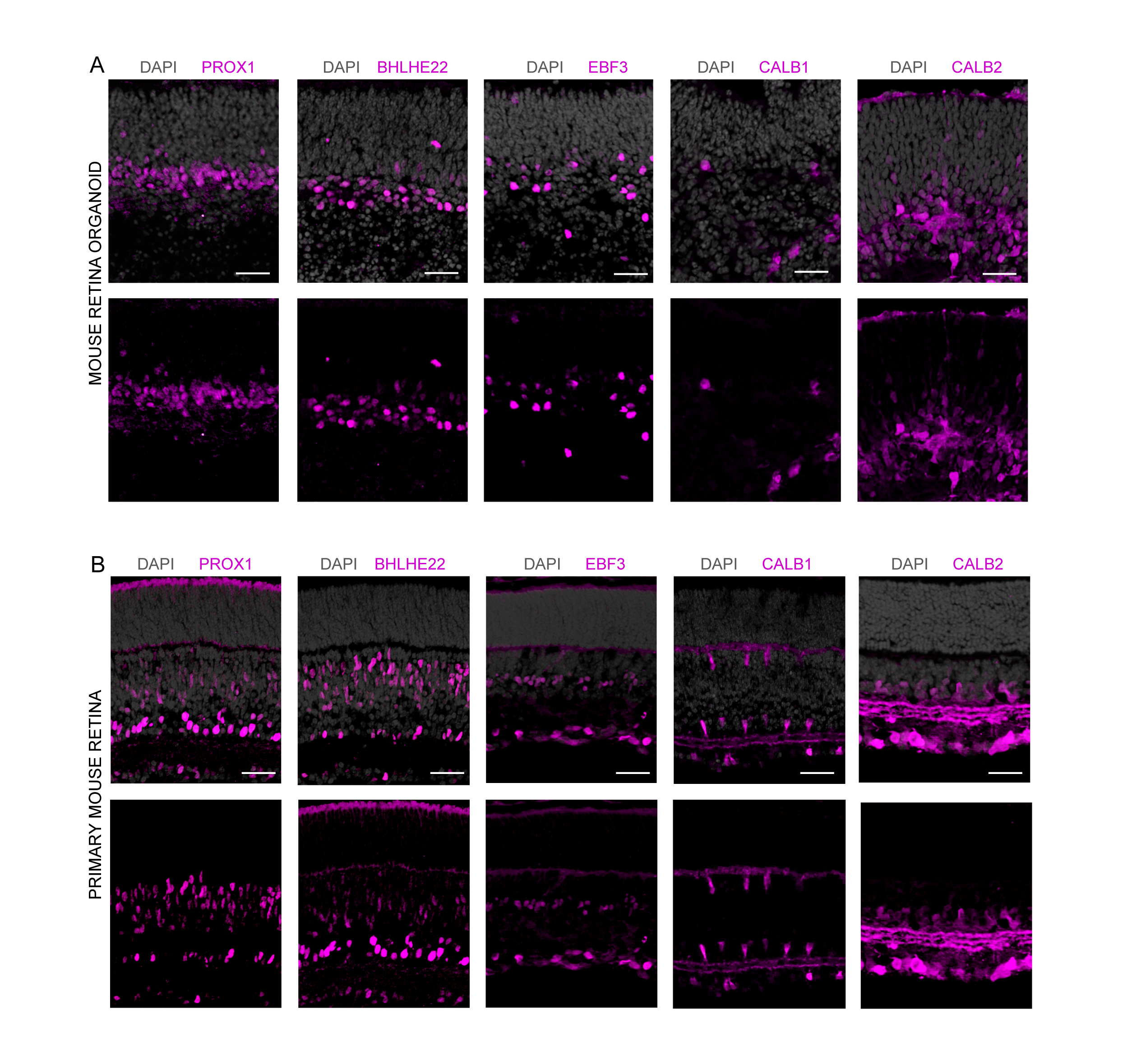

Supplement: Supplementary file 4 [file Image_1.TIF]

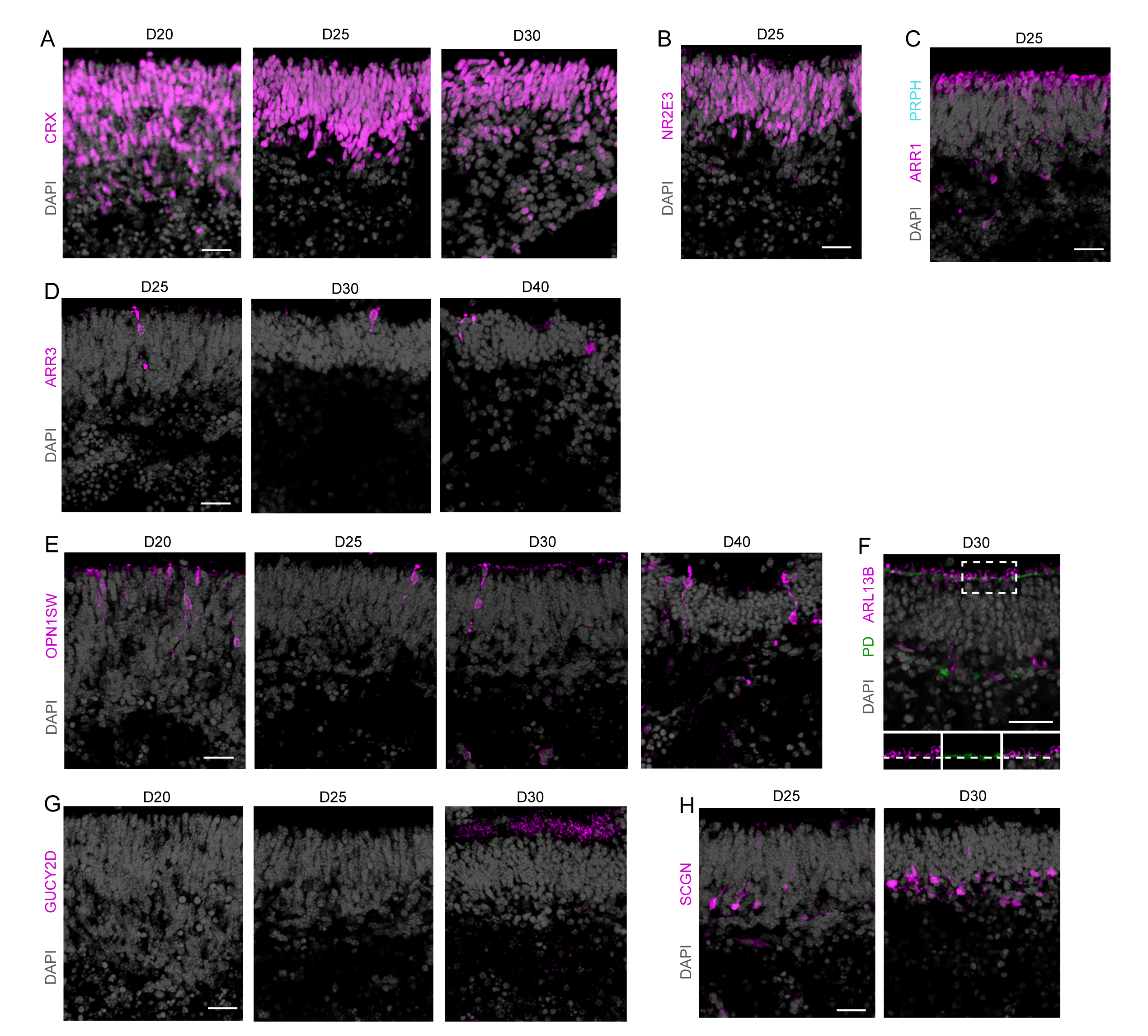

Supplement: Supplementary file 5 [file Image_2.TIF]

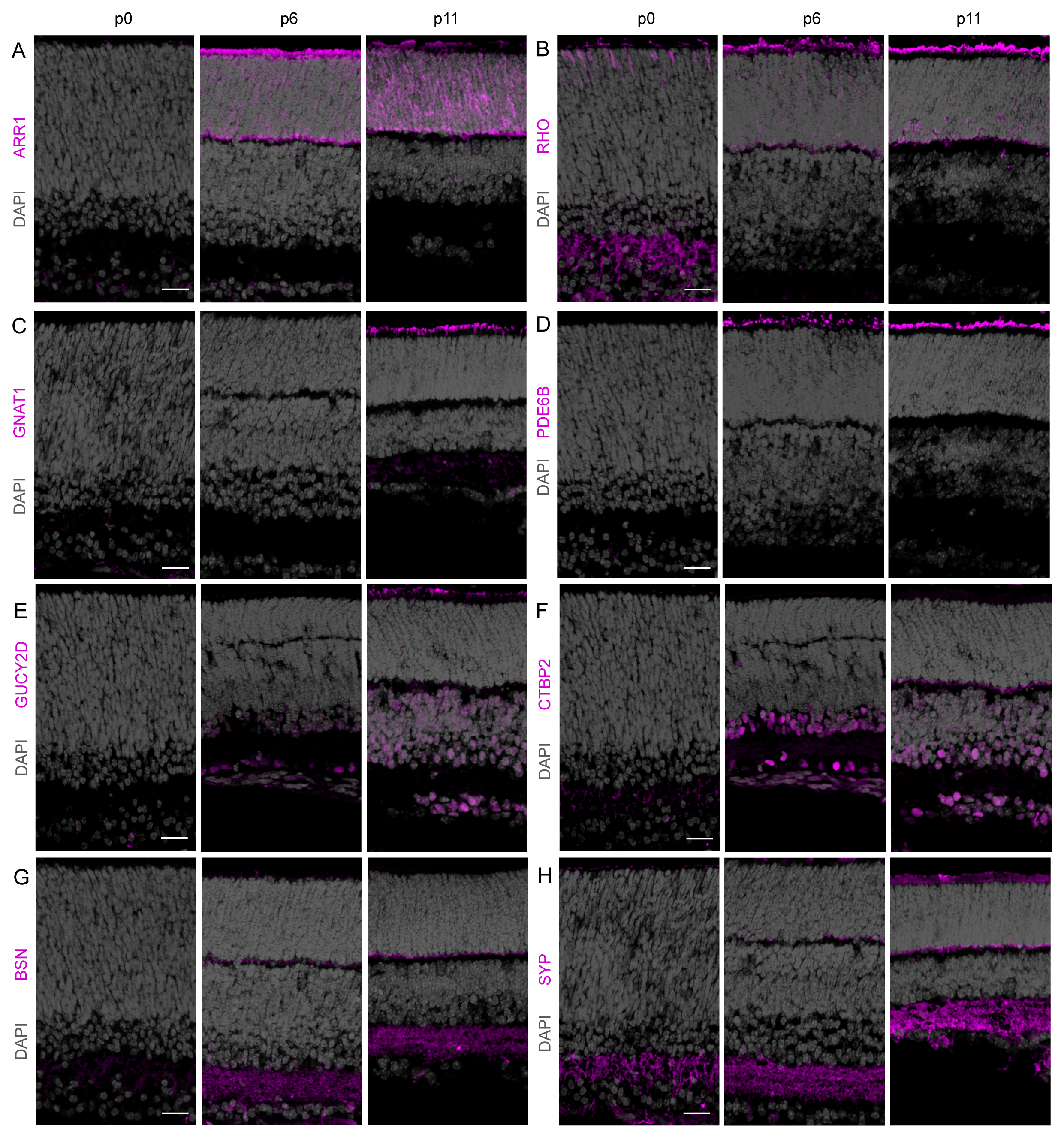

Supplement: Supplementary file 6 [file Image_3.TIF]

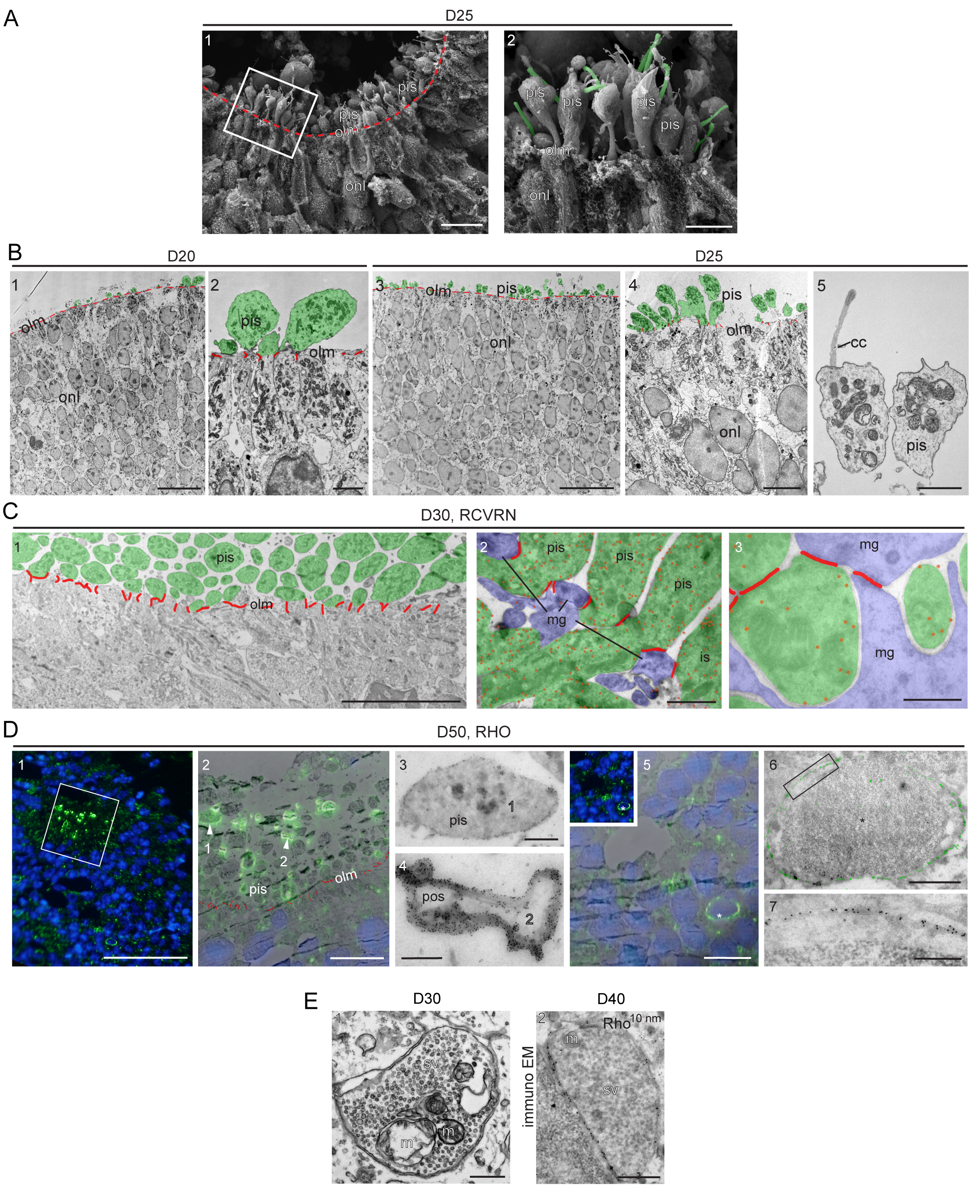

Supplement: Supplementary file 7 [file Image_4.TIF]

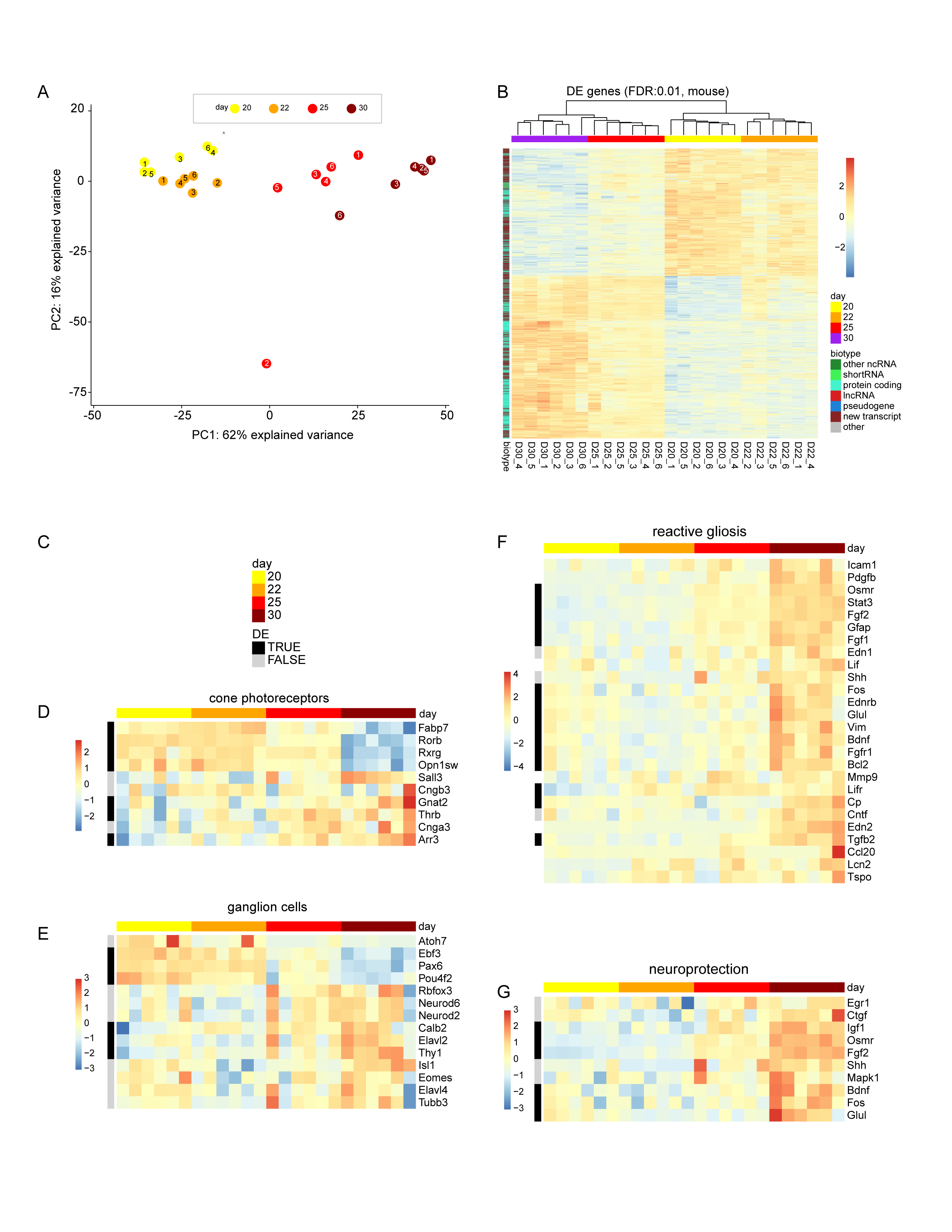

Supplement: Supplementary file 8 [file Image_5.TIF]

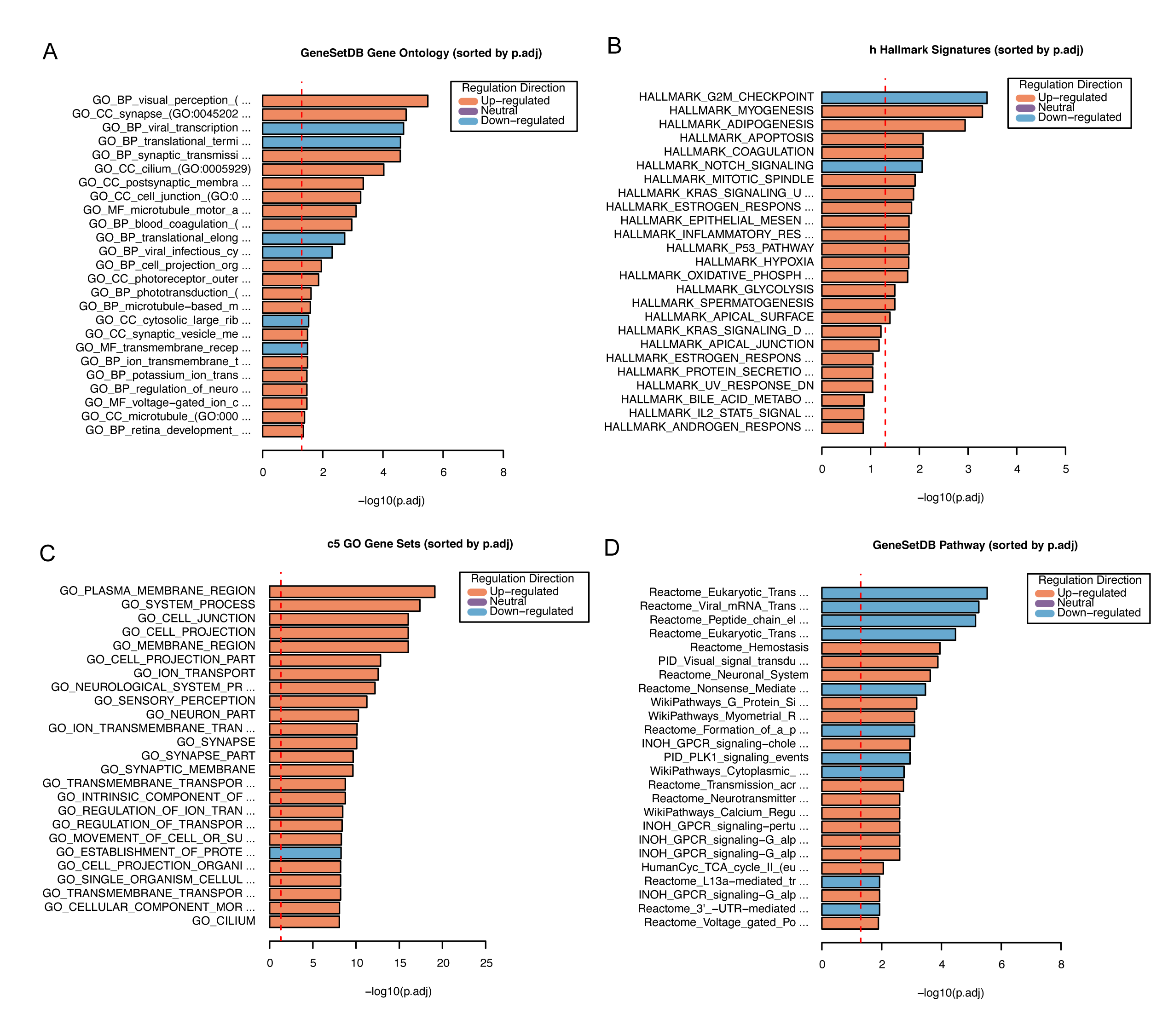

Supplement: Supplementary file 9 [file Image_6.TIF]

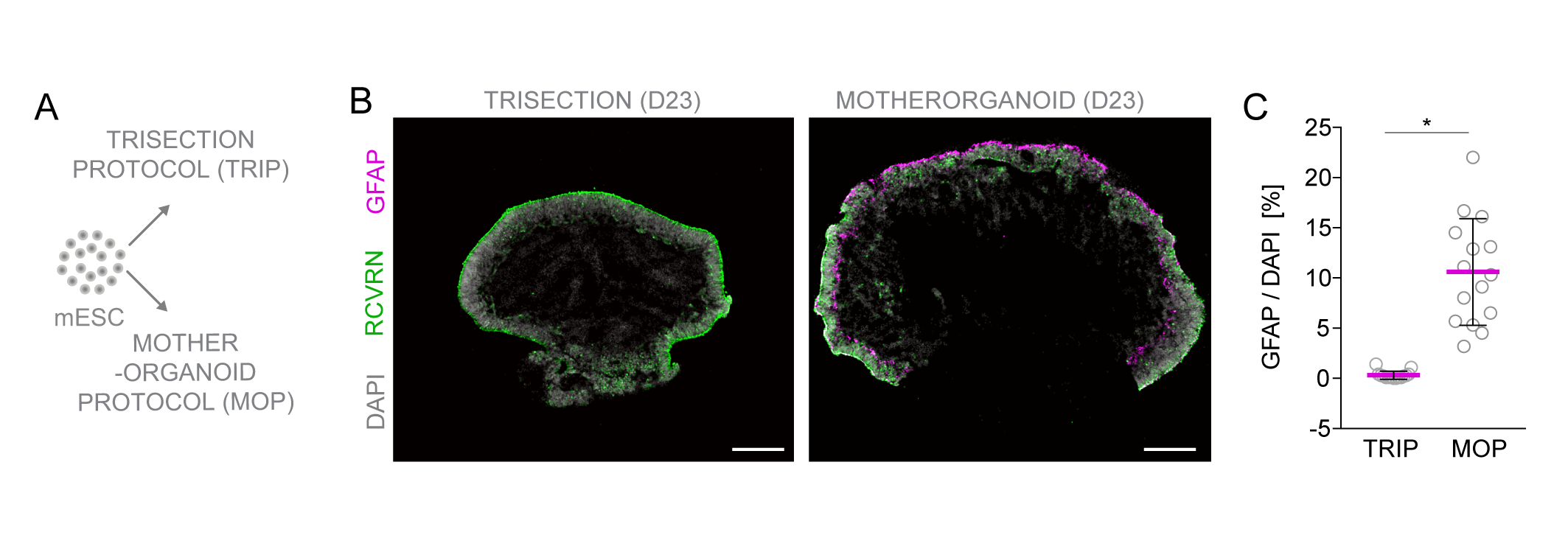

Supplement: Supplementary file 10 [file Image_7.TIF]

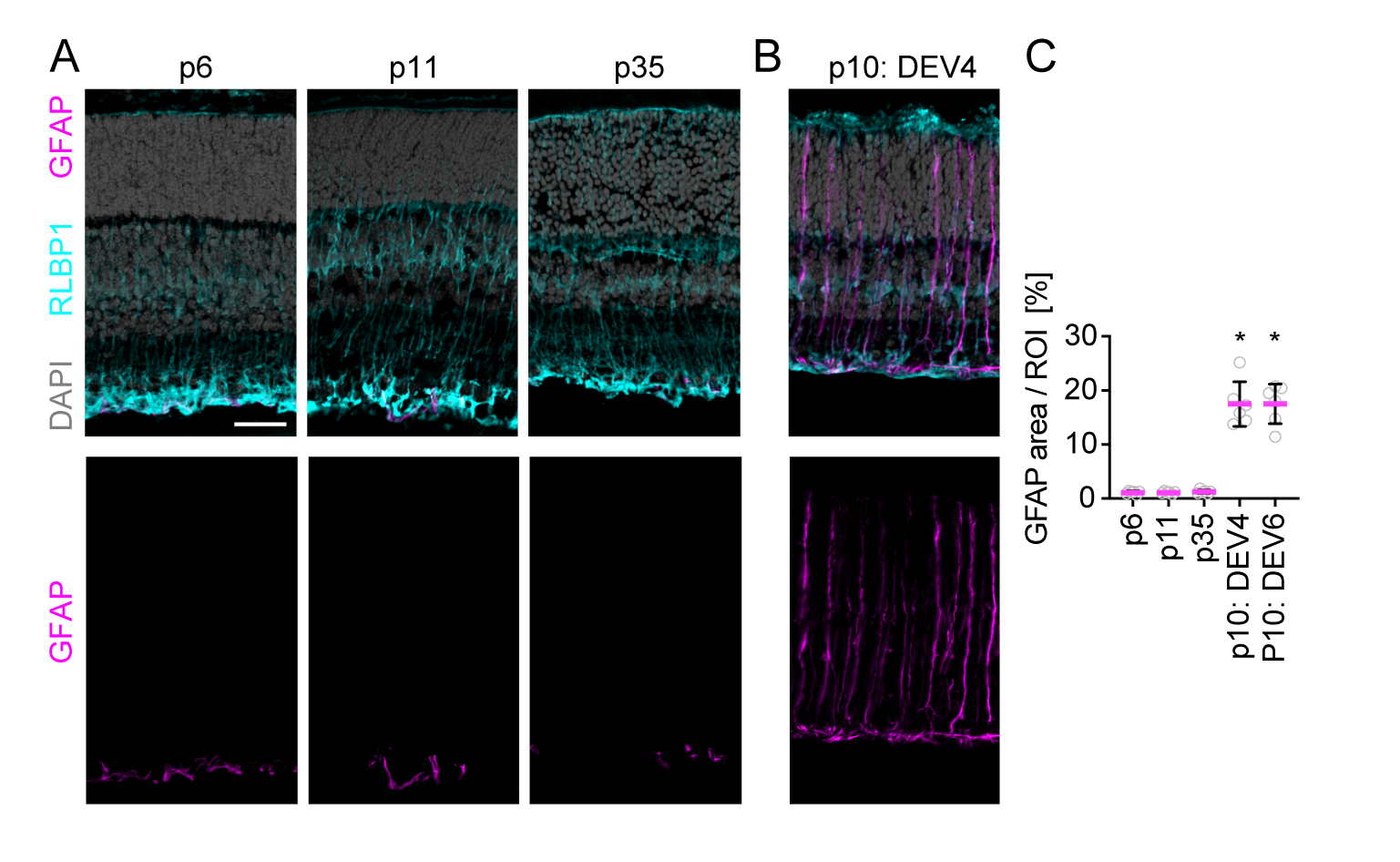

Supplement: Supplementary file 11 [file Image_8.TIF]

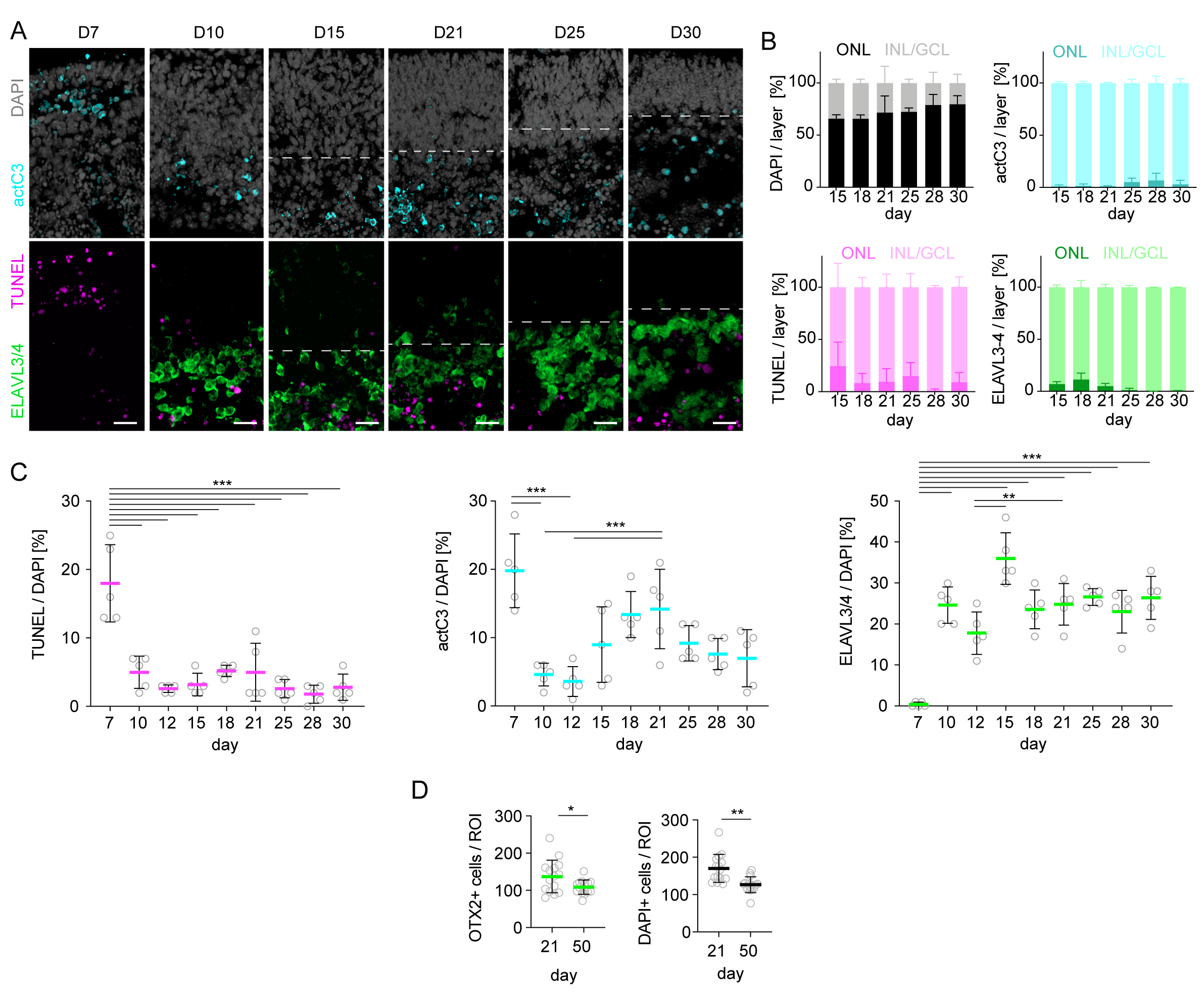

Supplement: Supplementary file 12 [file Image_9.TIF]

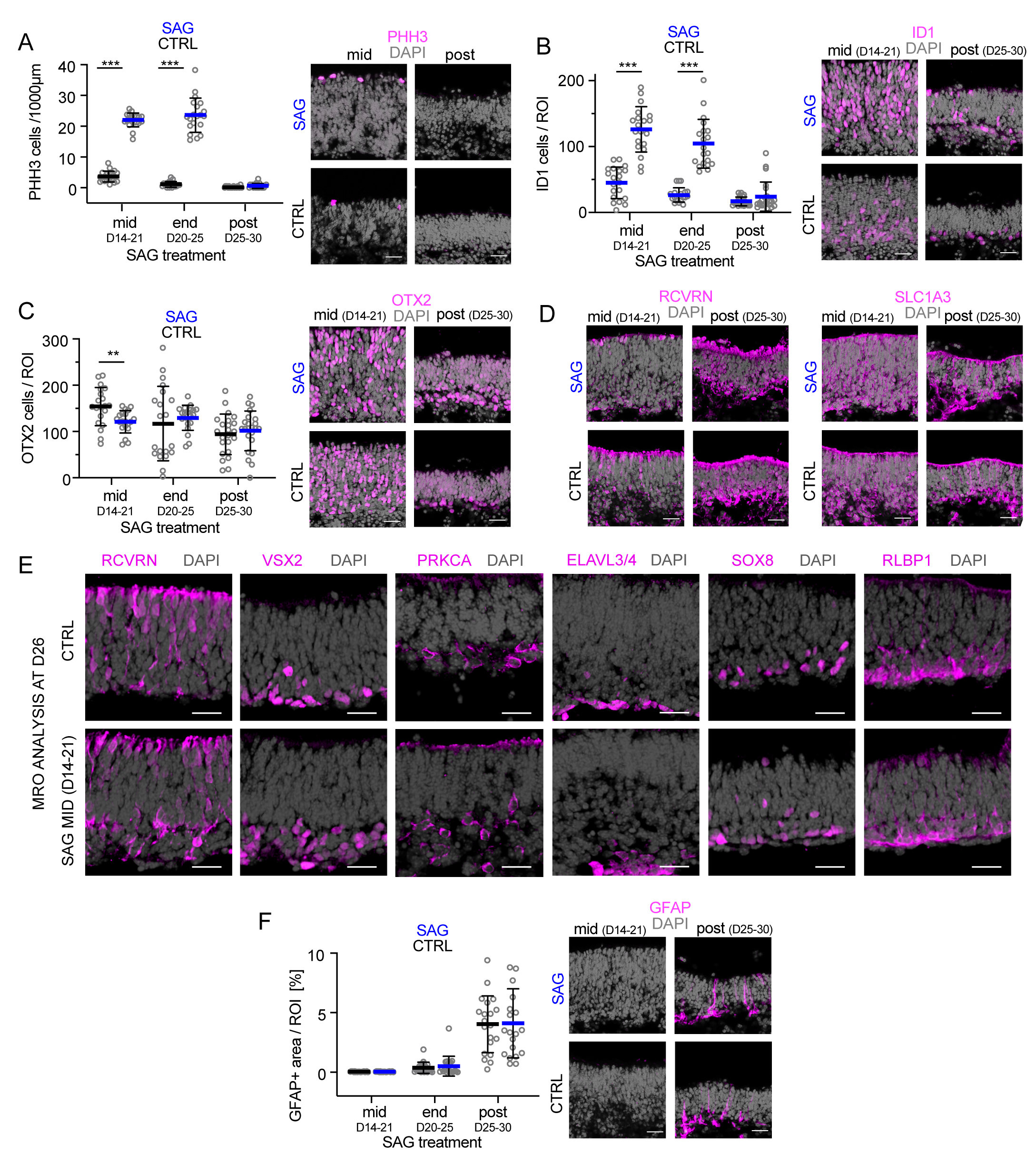

Supplement: Supplementary file 13 [file Image_10.TIF]

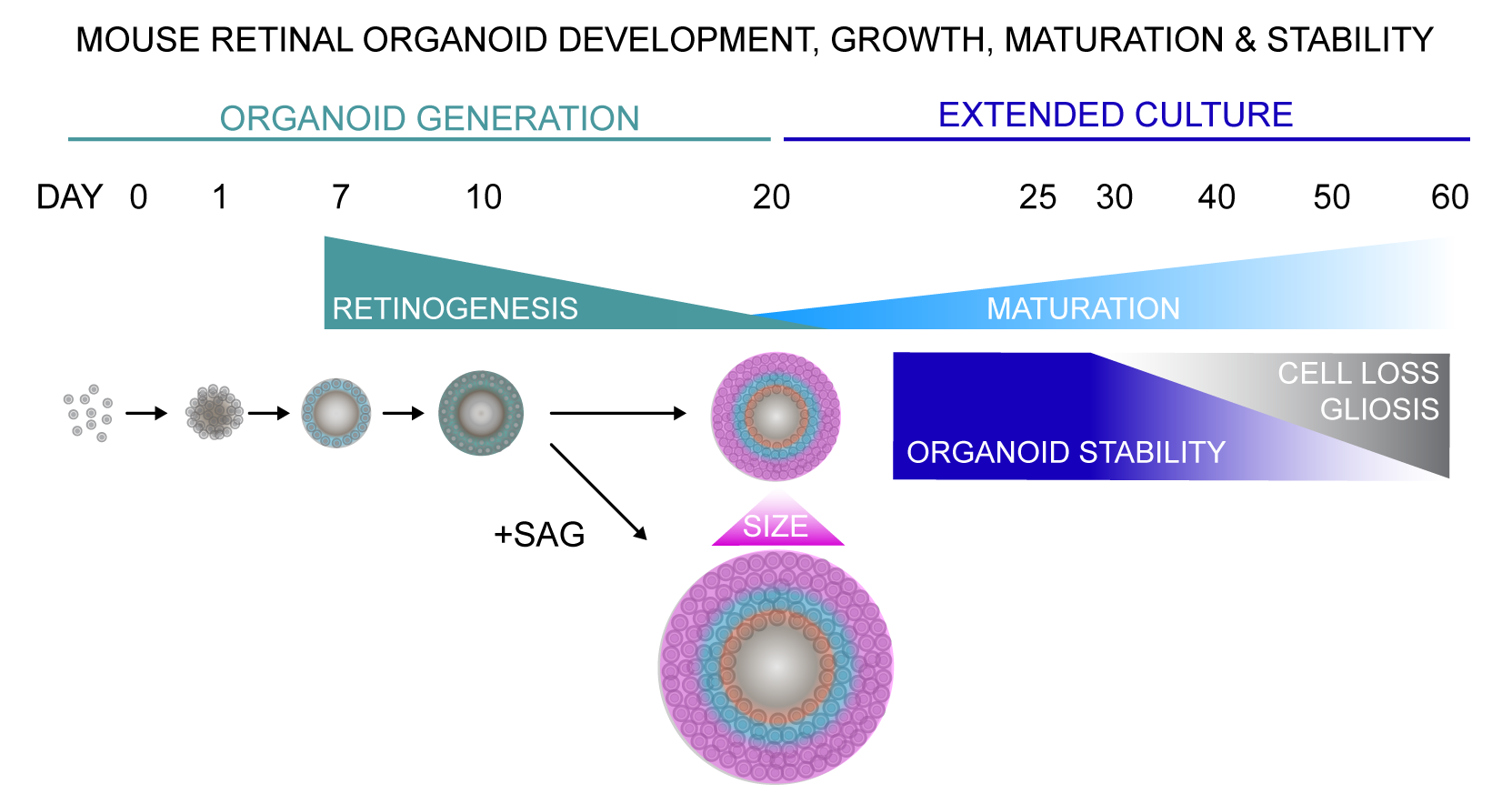

Supplement: Supplementary file 14 [file Image_11.TIF]
